# Photophysical behaviour, solvatochromism, and silver nanoparticle-induced superquenching of (E, E)-2,5-Bis(3,4,5-trimethoxystyryl) pyrazine with DFT calculations

**DOI:** 10.1038/s41598-025-20289-y

**Published:** 2025-09-29

**Authors:** Ehab A. Okba, Yomna M. Hanafi, Tarek A. Fayed, Mahmoud A. S. Sakr, Samy A. El-Daly

**Affiliations:** 1 Chemistry Department, Faculty of Science, Tanta University, Tanta, 31527 Egypt; 2 Center of Basic Science, Misr University for Science and Technology, 6TH of October City, Cairo, Egypt

**Keywords:** Photophysics, Dipole moment, Laser dye, Silver nanoparticles, Fluorescence quenching, DFT, Chemistry, Photochemistry, Physical chemistry

## Abstract

The fluorescence quantum yield (ϕ_f_), electronic transition dipole moment (µ_12_), molar absorptivity (ε), and fluorescence lifetime (τ_f_) of (E, E)-2,5-Bis(3,4,5-trimethoxystyryl) Pyrazine (BTMSP) were studied in various mediums. The medium’s polarity significantly affects fluorescence spectra, showing that the BTMSP is more polar in an excited state. Exciton emission was found in solid crystals of BTMSP at 550 nm upon excitation at 400 nm. The dipole moments of the ground and excited states were evaluated based on the Stokes shift and solvent polarity, showing a higher polarity of 6.22 Debye for the excited state than 2.02 Debye for the ground state. pKa and pKa* values for BTMSP have been determined as 1.95 and 10.88, respectively, indicating the higher basicity of the excited state. The green fluorescence (462–525 nm) was caused by the laser activity of BTMSP dye (0.5 mM) in DMSO, DMF, CH_3_CN, and dioxane when excited at 337.1 nm by a pulsed nitrogen laser. Fluorescence superquenching of BTMSP was detected using silver nanoparticles (AgNPs) in dynamic and static pathways. The Geometric and electrical structures of BTMSP dye were investigated using DFT methodologies. The outcomes of the experiments and the hypothesis were both consistent. The emission spectra of BTMSP are also studied in cationic and anionic surfactants to determine the critical micelle concentration.

**Supplementary Information:**

The online version contains supplementary material available at 10.1038/s41598-025-20289-y.

## Introduction

Organic dyes bearing electron-donating and electron-accepting substituents within the same molecular framework exhibit intriguing optical and spectroscopic properties. These donor–acceptor systems are of considerable interest due to their ability to undergo intramolecular charge transfer (ICT), a process in which electronic charge is transferred from the donor to the acceptor moiety. ICT can occur both in the ground state, forming a charge-transfer complex with distinct absorption features, and in the excited state, which significantly influences the dye’s fluorescence and overall photophysical behavior^1,2^.

ICT-active dyes often display substantial solvent-polarity-dependent changes in their photophysical characteristics. These include pronounced red shifts in emission spectra, significant variations in fluorescence quantum yield, and changes in excited-state lifetimes with increasing solvent polarity^3–5^. Such sensitivity to the surrounding medium makes ICT dyes highly valuable in chemical sensing^6^ (e.g., detecting solvent polarity or medium acidity), as well as in applications related to nonlinear optics and photoelectronic systems^7,8^.

Dye lasers have become indispensable tools in spectroscopy, photophysics, photochemistry, photobiology, the initiation of photochemical reactions, and numerous other scientific disciplines. In the past, the identification of organic dyes with the ability to demonstrate laser activity was carried out using a trial-and-error approach. Countless experimental investigations have been conducted to successfully generate stimulated emission using organic dyes^9–12^. The desirable attributes of a laser dye encompass a substantial molar extinction coefficient at the excitation wavelength, a broad emission band, less overlap between the emission band and triplet-triplet absorption band^11^notable photostability, and a brief duration of the excited state.

In parallel, metallic nanoparticles (MNPs), particularly silver (AgNPs) and gold nanoparticles (AuNPs), have garnered significant interest due to their unique physicochemical properties arising from high surface area-to-volume ratios. These include distinctive optical behaviors linked to surface plasmon resonance (SPR), which manifests as strong absorption in the visible region. Such properties make MNPs highly valuable in sensing, imaging, and therapeutic applications.

Significantly, several MNPs have demonstrated the ability to either quench or enhance the fluorescence of organic dyes, depending on factors such as distance, orientation, and local environment^13–15^. Gold nanoparticles have been reported to induce superquenching of acridinium ester^16^Rhodamine 6G^17^, and coumarin 153^18^. Silver nanoparticles also exhibit a significant quenching effect on various fluorophores, including fluorescein dye^19^Rhodamine 6G, and 4-hydroxycoumarin^20^. Additionally, zinc oxide and copper oxide nanoparticles have been shown to quench the fluorescence of chlorophyll^21^. On the other hand, nanoparticles of gold (Au), silver (Ag), and aluminium (Al) can enhance fluorescence in certain dye derivatives^22–27^.

There are primarily three ways that quenching can occur: statically, dynamically, and via electron/energy transfer quenching. An excited fluorophore undergoes collisions or interactions with the quencher in dynamic quenching, forming a complex that lacks fluorescence. On the other hand, static quenching involves the adsorption of dye molecules onto metallic nanoparticles with a non-fluorescent interaction between the fluorophore and the quencher(such as some perylene derivatives with silver nanoparticles^28^. Fluorescent quenching of coumarin 153 by gold nanoparticles^29^. Superquenching of organic dyes is often characterised by higher Stern-Volmer constants in the range of 10^7^–10^11^ mol^−1^ dm^3^ for metal nanoparticles. The process of energy transfer occurring between the dye molecules and the metal nanoparticles, resulting in the production of a nonfluorescent combination, may explain the superquenching of fluorescence by utilising metal nanoparticles^30^.

Fluorescence quenching of laser dyes by noble metal nanoparticles (NPs) has gained significant attention due to its role in enhancing various nanotechnology applications. This phenomenon is crucial in improving the performance of dye lasers, lithography, and biosensors, and is widely used in protein labeling, targeted cancer therapy, and molecular diagnostics. It also supports innovations in energy conversion, bio-imaging, and microbiological assays, where precise fluorescence control enables greater sensitivity and functionality^31^.

The present study aims to investigate the spectral and photophysical properties of the donor, comprehensively acceptor–type styryl pyrazine compound (E, E)−2,5-Bis(3,4,5-trimethoxystyryl)pyrazine across various media. The research further explores fluorescence quenching behavior in ethanol induced by colloidal silver nanoparticles (AgNPs) through steady-state emission spectroscopy. Additionally, the compound’s potential for laser activity and photostability is assessed. Complementary density functional theory (DFT) computations are also conducted to support and rationalise the experimental observations.

## Materials and methods

### Materials

The chemicals utilized in this research were sourced from Sigma Aldrich, guaranteeing an exceptional purity level of 99.8% and were utilized without undergoing additional purification processes. The characterization of BTMSP was accomplished using the Gallenkap melting point equipment and an FT-IR spectrophotometer (Shimadzu-8400 S) applying the KBr pellet method. Burker DPX-600 and 150 MHz NMR spectrometers were utilized for the collection of ^1^H and ^13^C NMR spectra, respectively. Both spectra employ tetramethyl silane as an internal standard. The chemical shift values are recorded on the δ scale and coupling constant(J) in Hz. Splitting patterns were designated as follows: s: singlet, d: doublet, m: multiplet. The electronic absorption spectra were taken in a controlled environment at room temperature using a Shimadzu UV-3101 PC spectrophotometer. At 298 K, a Jasco FP8200 spectrofluorometer was used to collect the fluorescence spectra. The fluorescence quantum yield (ϕ_f_) were calculated by comparing experimental results to those from the quinine sulphate standard. When quinine sulfate was dissolved in 0.5 mol dm^−3^ H_2_SO_4_, its fluorescence quantum yield (ϕ_f_) was measured to be 0.55^32,33^. To decrease the amount of reabsorption, minimum sample concentrations (0.1 absorbance unit) were used. We compared the fluorescence quantum yield to quinine sulphate using this Eqs^34^^35^.,

1 $$\phi\:{}_{\varvec{f}}\left(s\right)=\phi{}_{\varvec{f}}\left(r\right)\times\:\frac{\int\:{I}_{s}}{\int\:{I}_{r}}\times\:\frac{{A}_{r}}{{A}_{s}}\times\:\frac{{n}_{s}^{2}}{{n}_{r}^{2}}$$

A_r_ and A_s_ are the absorbances at the excitation wavelengths of the reference and sample, and n_r_ and n_s_ are the solvent’s refractive indices of the reference and sample, respectively The $\:\int\:{I}_{s}\:\:\:and\:\int\:{I}_{r}\:$ represents the area under the emission band for the sample and the reference, respectively. We characterized the silver nanoparticles using transmission electron microscopy (TEM) on a JEM 2100 instrument, operating at an accelerating 200 kilovolts (kV). The experimental procedure involved placing samples onto Cu grids (200 mesh) covered with a carbon layer. Using fluoroHub, a device developed by Horiba Scientific, we determined the fluorescence decay patterns in picoseconds using the single photon counting method. We assessed the lifetime using the Fluofit software integrated with the equipment. A nitrogen laser (GL-3300 PTI) was used to excite a dye laser (GL-302 Dye Laser PTI) to measure the laser emission. Pulse parameters include 1.48 mJ of energy, 800 ps of time, and 337.1 nm of excitation wavelength. We assessed the dye laser’s narrow-band output (ED 100, Gen-Tec) by employing a pyroelectric Joule meter. A secondary pyroelectric joule meter was employed to ascertain the pump laser’s output power (ED 200, Gen-Tec).

### Synthesis of BTMSP dye

**Scheme 1:**
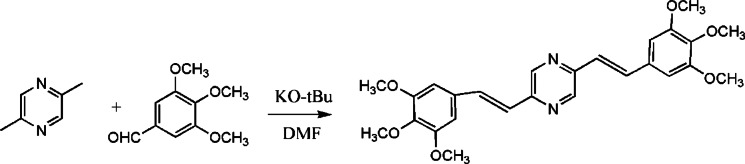
Synthesis of linear 2,5-bis (3,4,5-trimethoxystryl) pyrazine (BTMSP).

A mixture of 2,5-dimethylpyrazine (1.0 mmol), 3,4,5-trimethoxybenzaldehyde (5.1 mmol), and potassium tert-butanolate (2.4 mmol) was mixed in 10 mL of N, N-dimethylformamide as shown in Scheme 1. The resulting mixture was stirred and cooled under a nitrogen atmosphere to 0.0^◦^C. Subsequently, trace quantities of KO-tBu were added. The mixture reached the surrounding temperature, and the BTMSP dye formation was verified using thin-layer chromatography (TLC). The isolation of the final product was carried out through a series of extractions using chloroform after the addition of water^36^. A yellow solid of 150 mg (33% yield) was formed and characterised by a melting point of 219 °C. The structure of the BTMSP dye was confirmed through the analysis of ^1^H and ^13^C-NMR spectra. In the ^1^H-NMR spectrum (Fig. S1a) (600 MHz, CDCl_3_), the following chemical shifts were observed: δ = 3.76 (s, 6 H, 2OCH_3_ in position 4), 3.810 (s, 12 H, 4OCH_3_ in position 3,5), 6.91 (s, 4 H, Ph), 7.15–7.7 (m., 4 H vinyl proton), 8.65 (s, 2 H, Pyrazine ring).

Similarly, the ^13^C-NMR spectrum (Fig. S1b) (150 MHz, CDCl_3_) displayed the following carbon signals: δ = 56.08(C-1), 56.11(C-2). 100(C-5), 110.18 (C-8), 112.34 (C7), 121.78((C-6), 122.88(C-3), 129.67(C-10), 133.96(C-9), 143.61(C-4). The infrared (IR) spectrum obtained using KBr revealed characteristic peaks at ν/cm^−1^ = 3433, 2933, 1631, 1582, 1464, 1450, 1420, 1322, 1275, 1238, 1128, 1004, 965, 804, 640.

### Synthesis and characterization of silver nanoparticles

**Fig. 1:**
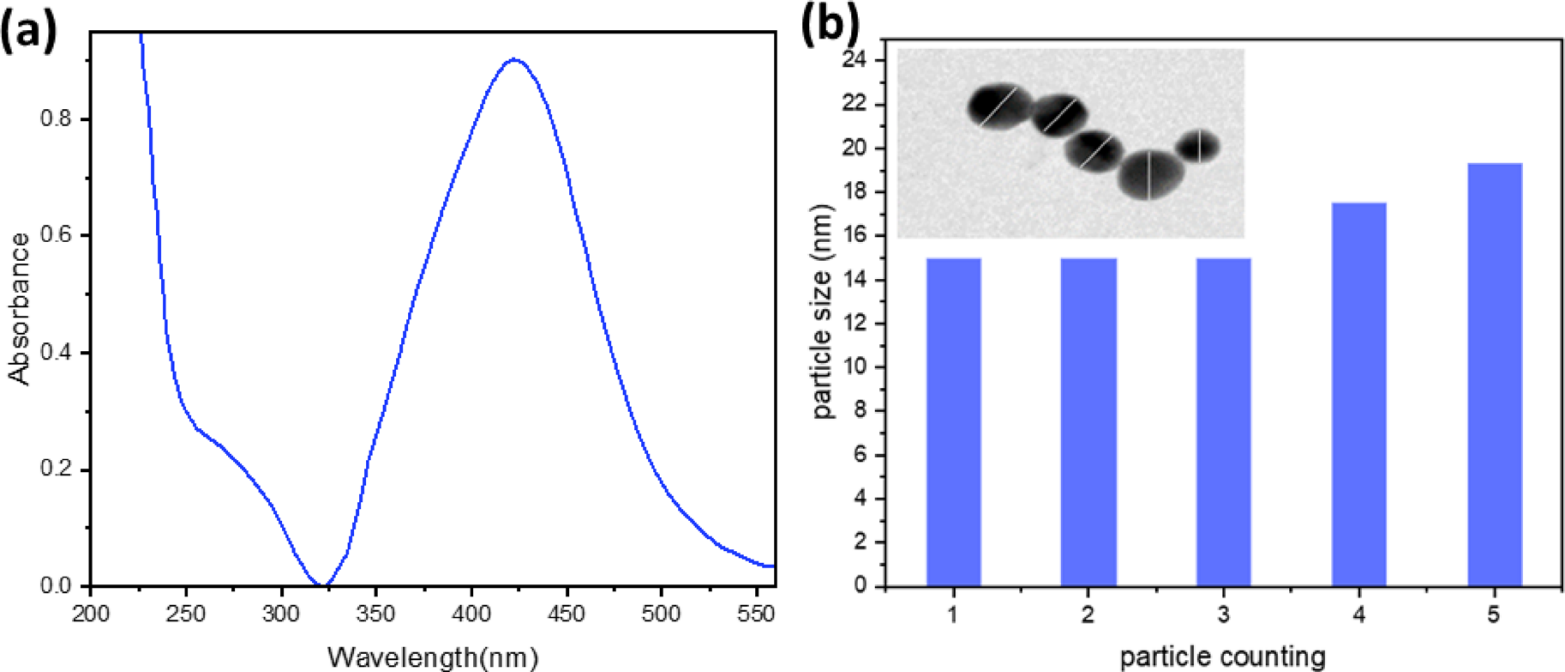
Absorption spectrum of 0.3 × 10^−9^ mol dm^-3^ AgNPs aqueous solution (**a**), histogram of Particle size against particle counting, inset; TEM images of the as-prepared AgNPs (**b**).

A silver nanoparticle was prepared by boiling a solution of AgNO_3_ (100 ml, 1 × 10^−3^ mol dm^-3^) with trisodium citrate (5 ml of 1%) as a reducing and nucleating agent. The solution’s colour was altered from yellow to golden yellow, showing a surface Plasmon band at 430 nm, signifying the successful synthesis of AgNPs as demonstrated in Fig. 1. The mixture was agitated until it reached ambient temperature. The transmission electron microscope (TEM) and ultraviolet-visible absorption spectra were used to characterise the silver nanoparticles. As depicted in Fig. 1, the silver nanoparticles have spherical shapes and diameters ranging from 20 to 30 nm.

## Results and discussion

### Effect of Solvent

**Fig. 2:**
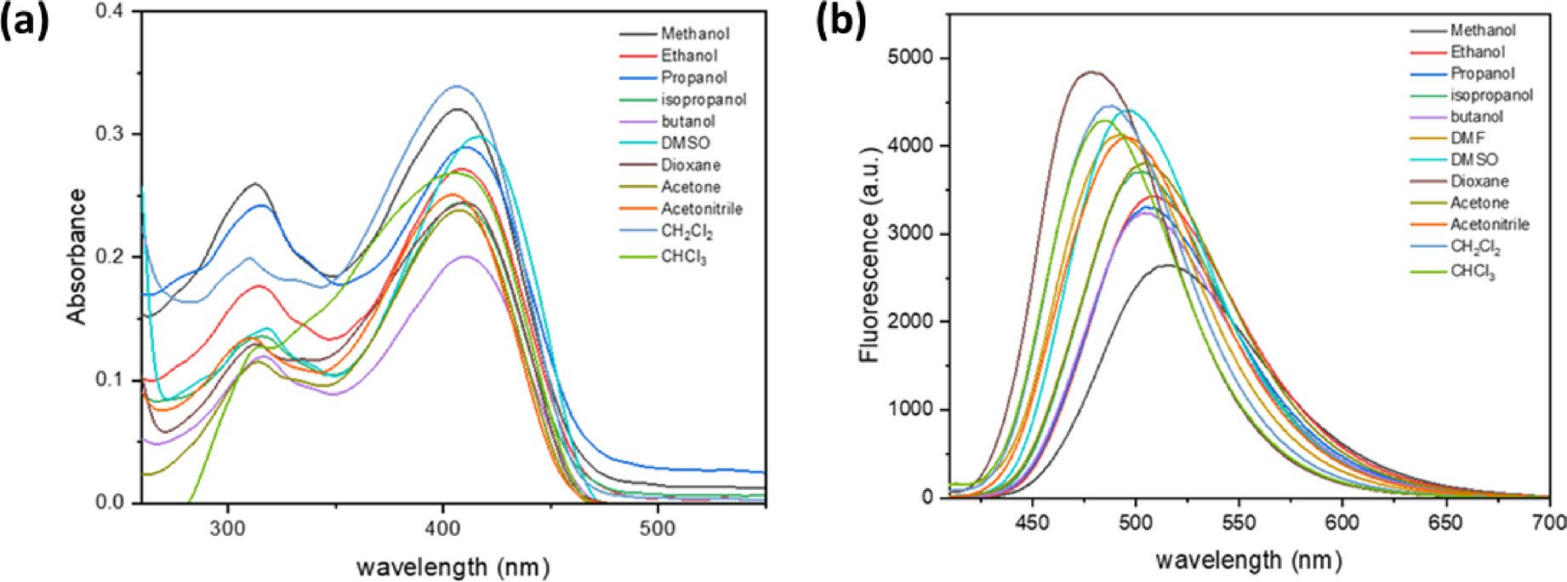
Electronic absorption spectra of BTMSP (10 × 10^−6^ mol dm^−3^) in various solvents (**a**) and Fluorescence spectra of BTMSP (5 × 10^−6^ mol dm^−3^) in different solvents at λ_ex_ = 410 nm (**b**).

**Fig. 3:**
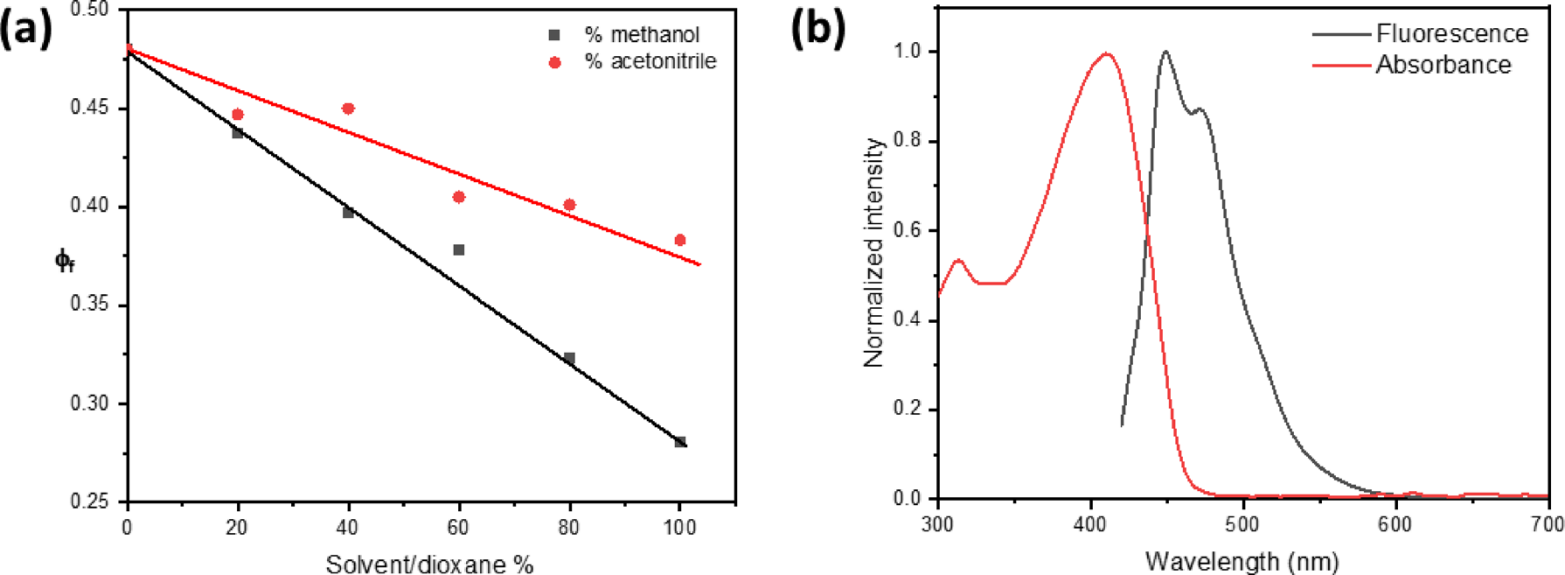
(**a**) Effect of CH_3_CN and methanol addition on fluorescence quantum yield of BTMSP in 1,4-dioxane (λ_ex_ = 400 nm). (**b**) The electronic absorption and fluorescence spectra of BTMSP dye in cyclohexane.

As shown in Fig. 2, the absorption spectra of a 1 × 10^−5^ mol dm^−3^ BTMSP dye solution were recorded at various solvent polarities. There is only a slight difference of about 6 nm between the maximum wavelength of the electronic absorption spectrum peaks as the polarity of the solvent increases. The results of this experiment point to a less polar ground state for BTMSP dye. Additionally, it exhibits a strong electronic absorption characterised by high molar absorptivity (ε) within the range of 238,000 to 339,000 dm^3^mol^−1^cm^−1^. a high oscillator strength (f) ranging from 0.400 to 0.89 at lower energy levels due to intramolecular charge transfer (ICT) in a very acceptable π -π* transition. On the other hand, the emission maxima undergo a red shift of 37 nm on increasing the solvent polarity from dioxane (478 nm) to methanol (515 nm) (Table 1 and S1), suggesting the excited state is more polar than the ground state. Thus, the emission spectra are more sensitive to solvent polarity than the absorption spectra, which indicates that a large charge transfer takes place in the excited state in comparison to the ground state. (Table 1 and S1). Increasing the solvent’s polarity E_T_ (30) causes a drop in the value of ***φ***_***f***_, as shown in Fig. S2. Solvent polarizability, hydrogen bonding, and other types of interactions are all included in the E_T_ (30) parameter for solvent polarity^1^. One explanation for the observed decrease in fluorescence intensity with increasing solvent polarity is the presence of highly excited polar ICT states. Furthermore, an essential non-radiative decay mechanism was detected in BTMSP, suggesting favourable solvatokinetic characteristics. The behaviour that has been noticed can be explained by the effective intersystem crossing and/or internal conversion, which is made easier by the significant mixing of the nearby (π-π*) and ^1^(n-π *) states^11,37–40^. Polar protic solvents show an increase in the non-radiative rate, which includes vibrational cascade, internal conversion, and intersystem crossing^5,41,42^. As a result, the fluorescence quantum yield (***φ***_***f***_) and excited-state lifetime (τ_f_) are lower in polar protic solvents, such as ethanol and methanol, than in other solvents. This is attributed to the formation of hydrogen bonds with the pyrazinyl moiety and the methoxy groups in BTMSP, which consequently opens channels for radiationless processes. One may verify this by examining how ***φ***_***f***_ varies in solvent mixtures containing dioxane-methanol and dioxane-acetonitrile. The fluorescence quantum yield decreases as we add more methanol or acetonitrile to dioxane. This happens more quickly with methanol than with acetonitrile because of hydrogen bonding, as shown in Fig. 3a.

In a non-polar solvent like cyclohexane, the fluorescence and absorption spectra of BTMSP are not identical (Fig. 3b). The appearance of two vibrational peaks in the emission spectrum, corresponding to (0 ← 0) and (1 ← 0) transitions, indicates that the planarity of BTMSP increases upon excitation. Generally, deviations from the mirror image rule often suggest noticeable differences in the geometric configurations of the nuclei between the excited and ground states.

**Table 1:** Photophysical parameters of BTMSP in different solvents.

Solvents	λ_max(Abs)_ (nm)	λ_max, f_ (nm)	Δν cm^−1^	ε_max_ dm^3^.mol^−1^ cm^−1^	τ_f_ _ns_	φ_f_
1,4 Dioxan	410	478	3469.7	247,000	1.60	0.48
CHCl_3_	404	484	4091.3	268,000	1.59	0.40
CH_2_ Cl_2_	404	486	4176.3	339,000	1.59	0.32
n-BuOH	410	505	4588.2	200,000	1.56	0.42
iso-prOH	407	502	4649.7	245,000	1.57	0.37
n-propanol	410	506	4627.3	290,000	--	0.29
Acetone	410	504	4548.9	238,000	--	0.40
EtOH	410	507	4666.3	272,000	1.45 1.32	0.32
MeOH	404	515	5335.0	339,000	1.12	0.20
CH_3_CN	405	495	4489.3	251,000	1.65	0.39
DMF	404	491	4385.8	296,000	1.48	0.47
DMSO	415	496	3935.0	298,000	1.53	0.42

The BTMSP crystal in its solid state emits strong yellow fluorescence with a peak cantered at 550 nm and a spectral width ranging from 63 to 73 nm when excited by light between 365 and 420 nm, as shown in Fig. S3a. Notably, the emission peak remains unchanged across this excitation range, confirming that the observed fluorescence is genuine and not a result of second-order diffraction effects^43–45^. This stable emission is attributed to excitonic states within the crystal, as evidenced by a significant red shift of 96 nm relative to a dilute BTMSP solution in cyclohexane (1 × 10^−5^ mol dm^−3^) and 25 nm compared to a thin film formed from acetone, Fig. S3b. These findings suggest that the crystal structure corresponds to the β-type configuration^46^. Due to their promising optical characteristics, solid-state luminescent materials like BTMSP have attracted growing interest for applications in advanced technologies, particularly in the development of light-emitting diodes (LEDs)^47^.

### Measurement of the BTMSP Dipole Moment

The dipole moment difference between the singlet excited and ground states can be determined by studying the solvatochromic effect. The solvent’s polarity directly impacts the Stokes shift, where a larger shift signifies a higher polarity of the singlet excited state relative to the ground state. Employing a streamlined rendition of the Lippert-Mataga equation, solvatochromic analysis offers a method to approximate the dipole moment difference (µ_e_-µ_g_) between the singlet excited and ground state^48,49^.

2 $$\:{\nu\:}_{a}-{\nu\:}_{f}={m}_{1}.\:{F}_{1}\left(\epsilon\:,n\right)+constant$$

Where 

3 $$\:{m}_{1}=\frac{2{\left({\mu\:}_{e}-{\mu\:}_{g}\right)}^{2}}{hc{a}^{3}}$$

4 $$\:{F}_{1}\left(\epsilon\:,n\right)=\left[\frac{\epsilon\:-1}{2\epsilon\:+1}-\frac{{n}^{2}-1}{2{n}^{2}+1}\right]$$

The Stokes shift, Δv_st_ (ν_a_ – ν_f_), increases as the polarity of the medium increases. The variables in the equation are as follows: h represents the Planck constant, c represents the light speed in space, (a) represents the radius of the Onsager cavity of the BTMSP, and ε and n represent the dielectric constant and refractive index of the solvent, respectively. This demonstrates that the excited state exhibits greater stability in polar liquids. Suppan’s equation refers to a certain mathematical Equation^50^. was applied to find the Onsager cavity radii (a) using the volume of molecules.

5 $$\:a={\left(\frac{3M}{4\pi\:\delta\:N}\right)}^{\raisebox{1ex}{$1$}\!\left/\:\!\raisebox{-1ex}{$3$}\right.}$$

**Fig. 4:**
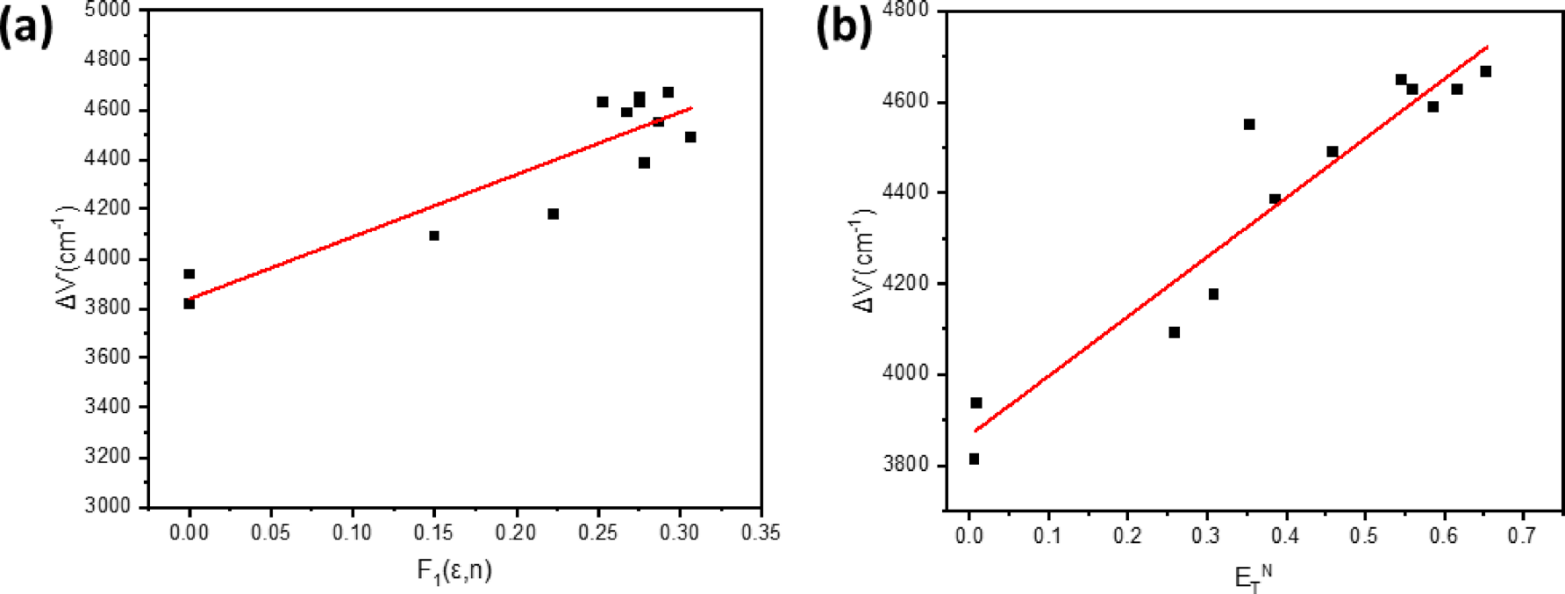
Variation of Stokes’ shift versus F_1_ (ε, n) (**a**), E_T_ (30) of different solvents (**b**).

δ represents the density of the dye, M stands for the molecular weight of the dye, and N represents Avogadro’s constant. Figure 4a illustrates the relationship between the Stokes shift and polarizability F_1_(ε,n), revealing that the BTMSP radius (a) is 4.2 Å. Hydrogen-bonding as a specific solute-solvent interaction was avoided by excluding data from polar protic solvents. From the slope, the change in dipole moment of BTMSP was calculated as 4.29 Debye upon excitation. This change in dipole moment is caused by the redistribution of atomic charges as a result of charge transfer from the electron-rich methoxy groups to the electron-deficient pyrazinyl moiety.

Using the dimensionless microscopic solvent polarity parameters $\:{E}_{T}^{N}$ given by Eqs. (6 and 7), The solvatochromic shift technique by Reichardt^1^ has been used to further analyse the discrepancy in dipole moment (Δµ) between the excited and ground states:

6 $$\:{E}_{T}^{N}=\frac{{E}_{T}\left(solvent\right)-30.7}{32.4}$$

7 $$\:{E}_{T}\left(solvent\right)=\frac{28591}{{\lambda\:}_{max}\left(nm\right)}$$

The term λ_max_ denotes the wavelength at which the intramolecular charge transfer absorption of the betaine dye reaches its pinnacle within the red region of the spectrum. The primary advantage of this strategy in comparison to the Lippert-Mataga method is that hydrogen bonding is now accounted for in the solvent parameter in addition to solvent polarity. The dipole moment change is determined using Eq. (8) (Fig. 4b).

8 $$\:{\Delta\:}\nu\:=11307.6{\left(\frac{{\Delta\:}\mu\:}{{\Delta\:}{\mu\:}_{D}}\right)}^{2}{\left(\frac{{a}_{D}}{a}\right)}^{2}{E}_{T}^{N}+constant$$

Where Δµ_D_ and Δµ are the dipole moment differences between the excited and ground states of betaine dye and BTMSP dye, respectively, and (a_D_) and (a) represent the radii of the Onsager cavity of betaine dye and BTMSP dye, respectively. Given that the value of a_D_ is 6.2 Å, a is 4.2 Å, and Δµ_D_ is 9 Debye. We can use Eq. (9) to get the resulting dipole moment change^51–53^.

9 $$\:{\Delta\:}\mu\:={\left[\frac{8lm{\times\:\left(\frac{6.2}{a}\right)}^{-3}}{11307.6}\right]}^{\frac{1}{2}}$$

In Fig. 4b, the slope of the linear plot of Stokes shift (Δµ) versus $\:{E}_{T}^{N}$ was determined, giving a value of 1.76 D for Δµ. However, when the Lippert–Mataga equation was applied. a higher value was obtained compared to that derived from the dimensionless microscopic solvent polarity parameter ($\:{E}_{T}^{N}).\:$This disparity arises because the Lippert–Mataga equation is only an approximation and neglects higher-order terms. Such terms may account for additional effects, including the induction of dipole moments in solvent molecules by the excited fluorophore, and vice versa^29^. On the other hand, the Lippert–Mataga equation yields a Δµ value of 4.29 D, which is close to the difference between µ_e_ and µ_g_ (4.3 D), as determined using Bakshiev’s and Kawski–Chamma–Viallet equations in the Supporting Data (Fig. S4).

#### Effect of medium acidity

**Fig. 5:**
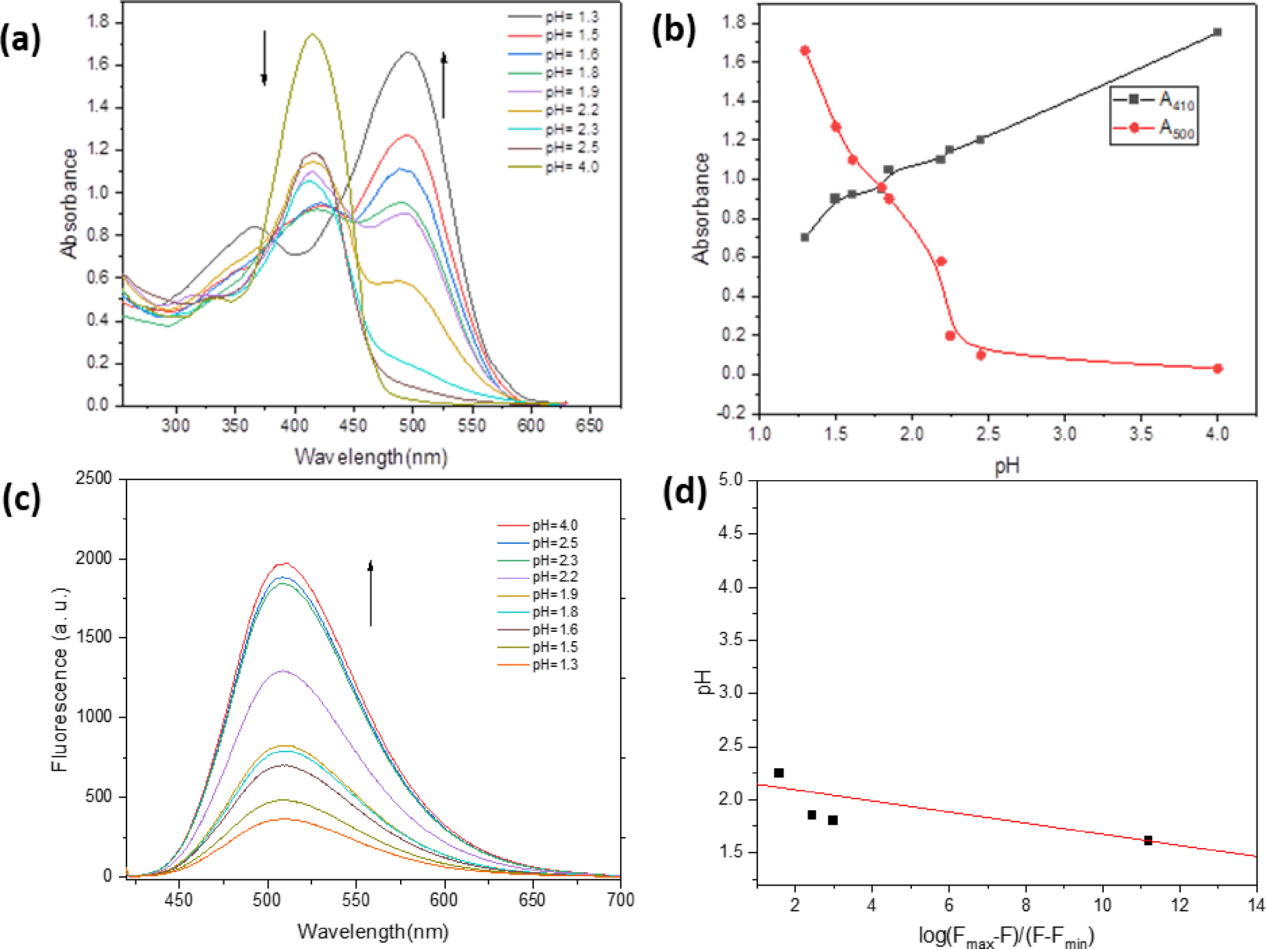
Absorption spectrum of BTMSP in different pH in ethanol-sulfuric acid (**a**), absorbance-pH measurements using the half-height method (**b**), emission spectrum of BTMSP in different pH in ethanol-sulfuric acid (**c**), Modified limiting emission method; pH versus log fluorescence ratio of dye at λ_flu_ = 530 nm and λ_ex_ = 400 nm (**d**).

The fluorescence and electronic absorption spectra of BTMSP have been investigated in various acidic environments in 40% ethanol by manipulating the concentrations of hydrogen ions in 0.5 mol dm^−3^ H_2_SO_4_ (Fig. 5). In an acidic solution, a Further absorption band emerges at a wavelength of 500 nm, accompanied by an isosbestic point observed within the range of 435–450 nm (Fig. 5a). In acidic media, fluorescence is not emitted as much and is instead quenched (Fig. 5c) (since the protonated form is no longer emitted). The protonation of heterocyclic nitrogen, followed by a shift in the dye’s absorption spectra in acidic conditions, is an apparent cause of this spectral pattern modification. The half-height method was used to get the ground-state protonation constant (pKa) from absorption measurement, as shown in Fig. 5b). In contrast, the modified limiting emission method was used to determine pK_a_, as shown in Fig. 5d). The pKa value according to absorption and emission measurements is 1.81 and 2.1, respectively. The average value of pKa is 1.95. The excited state protonation constant pK_a_∗ was calculated using the following relation^54,55^:

10 $$\:p{k}_{a}-p{k}_{a}^{*}=2.1\:x\:{10}^{-3}\left({\stackrel{-}{\nu\:}}_{{BH}^{+}}-{\stackrel{-}{\nu\:}}_{B}\right)$$

In acidic and conjugate bases, the wave number of the pure electronic transition is different by amounts. The pKa* value of 10.88 agrees with the finding that the S_1_ excited state is more basic than the ground state. Although the Förster cycle is frequently employed to estimate pKa*^56^, it assumes identical solvation and structural environments in ground and excited states, which is not strictly valid for BTMSP. For this reason, we adopted the modified limiting emission method, which provides a more reliable estimate of pKa* under our experimental conditions.

#### Photostability and photoreactivity of BTMSP

Prolonged irradiation of 1 × 10⁻⁵ mol dm^−3^ of BTMSP in DMSO, DMF, CH₃CN, Dioxane, and CH₃OH for 120 min at 365 nm (I₀ = 4.5 × 10⁻⁴ Einstein min⁻¹) resulted in no significant changes in absorbance, indicating the high photostability of BTMSP in these solvents.

Higher photostability is an essential requirement for applying biomedical fluorescent labels and lasers. The experiment on photostability was conducted under controlled conditions, at ambient temperature, while ensuring the absence of natural light.

**Fig. 6:**
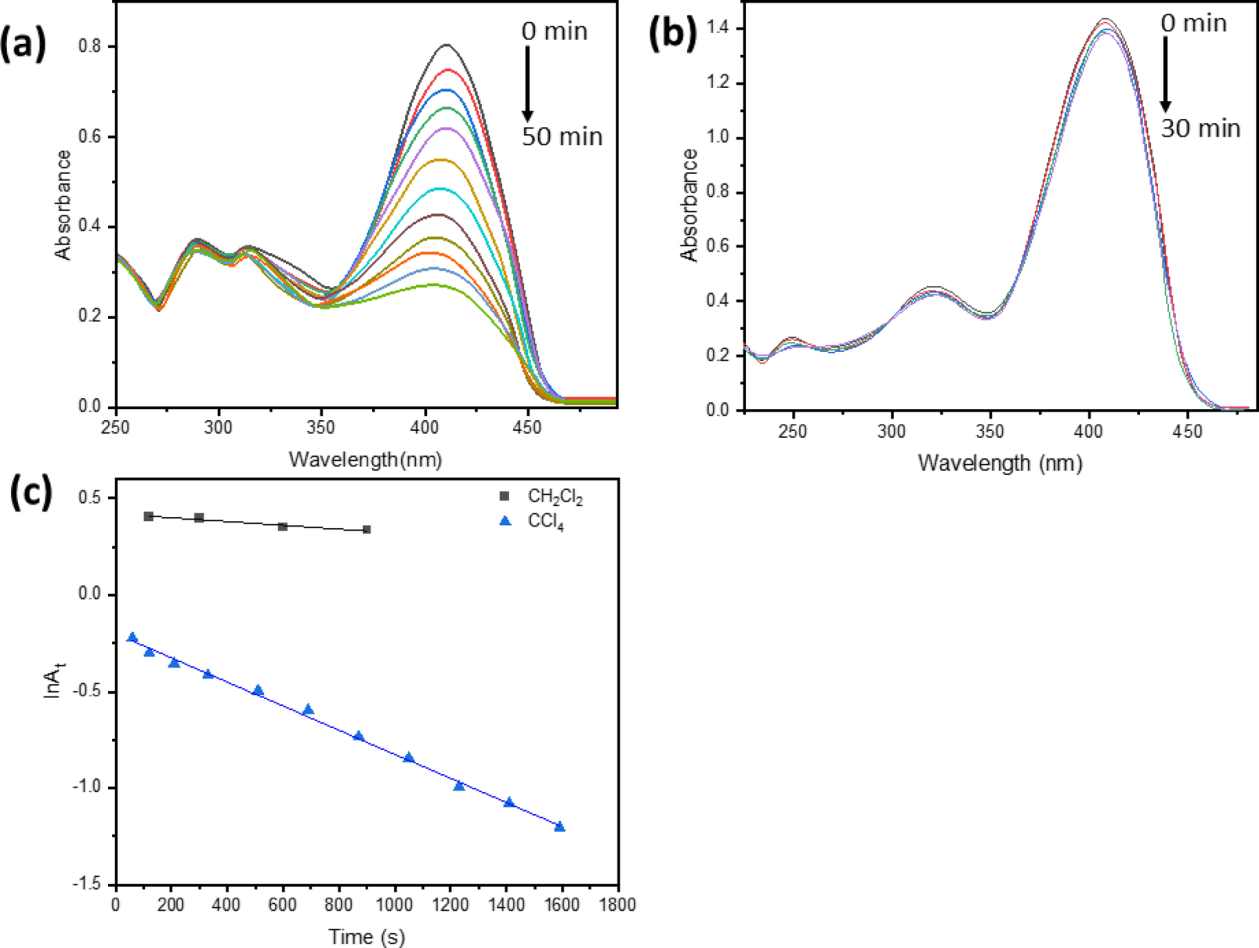
Absorption spectra of BTMSP in CCl_4_ against time, 2,4,6,8,12,14, 17,20,23,26,31,39, and 50 min (**a**), in CH_2_Cl_2_ against time 5,10,15, and 30 min (**b**), and first-order decomposition of BTMSP in CH_2_Cl_2_ and CCl_4_(**c**).

BTMSP solution (1 × 10^−5^ mol dm^−3^) in chlorinated solvents, specifically CH_2_Cl_2_ and CCl_4_, displays photodecomposition when exposed to radiation at a wavelength of 365 nm. The absorbance decreases until photostability is achieved, as illustrated in Fig. 6a,b. The rate constant associated with a photodecomposition can be determined by applying the first-order Eq. (11)^57^.

11 $$\:-\frac{d\left[BTMSP\right]}{dt}=k\left[BTMSP\right]\left[C{H}_{n}C{l}_{4-n}\right]$$

 

But $\:\left[C{H}_{n}C{l}_{4-n}\right]\gg\:\left[BTMSP\right]$, so $\:\left[C{H}_{n}C{l}_{4-n}\right]$ *is considered constant*

The equation becomes

12 $$\:-\frac{d\left[BTMSP\right]}{dt}=k\left[BTMSP\right]$$

13 $$\:\text{l}\text{n}{\left[BTMSP\right]}_{t}=ln{\left[BTMSP\right]}_{0}-kt$$

In the equation, [BTMSP]_0_ represents the initial concentration of BTMSP at time = 0, whereas [BTMSP]_t_ represents the concentration of BTMSP after a specified duration of illumination in minutes. According to Lambert-Beer’s law, a linear relationship exists between a substance’s concentration and its maximal absorption at low concentration. Eq.(14) can be interpreted as follows:

14 $$\:ln{A}_{t}=ln{A}_{0}-kt$$

The photodegradation reaction constant, k, is used in the formula alongside the maximum absorbance of the dye before and after illumination, A_0_ and A_t_, respectively. The rate constant of photodecomposition of BTMSP (k) in CH_2_Cl_2_ and CCl_4_ upon irradiation at 365 nm was determined from the slope of the first-order kinetic plot (Fig. 6c). The value of k is 0.09 and 0.6 × 10^3^ s^−1^, respectively. According to these findings, carbon tetrachloride is an excellent medium for the photoreactivity of BTMSP.

The photodecomposition of BTMSP in chloromethane, CH_n_Cl_4−n,_ seems to be based on a well-known process. It involves the transfer of electrons from excited BTMSP to chloromethane^58–65^. The basic photochemical mechanism has been postulated to include electron transfer from the excited singlet BTMSP^*^ to the chlorinated solvent within a transient excited charge transfer complex (exciplex, step III). It produces a chloride ion, a chloromethyl radical, and a BTMSP radical cation in a solvent cage (step V). For example, CH_2_Cl_2_ and CCl_4_ both have an electron affinity (EA) of 1.36 and 2.12 eV, electrons must therefore move from an excited donor to an electron acceptor for a pair of contact ions to be formed^58,64^. This indicates that the electron affinity of the solvent plays a crucial role in influencing photoreactivity and controlling the photochemical reaction. The formation of a photoproduct is a one-photon process as represented by equations I – V.

I $$\:\left(TMDSP\right)\:\left({S}_{0}\right)+h\upsilon\:\to\:{\left(TMDSP\right)}^{*}\:\left({S}_{1}\right)$$

II $$\:{\left(TMDSP\right)}^{*}\:\left({S}_{1}\right)\:\:\:\to\:\left(BTMSP\right)\:\left({S}_{0}\right)\:\:\:+{\left(h\upsilon\:\right)}_{Flu}$$

III $$\:{\left(TMDSP\right)}^{*}\:\left({S}_{1}\right)+C{H}_{n}C{l}_{4-n}\to\:{\left[TMDSP\dots\:..C{H}_{n}C{l}_{4-n}\right]}^{*}\left({S}_{1}\right)$$

IV $$\:{\left[TMDSP\dots\:..C{H}_{n}C{l}_{4-n}\right]}^{*}\underrightarrow{electron\:transfer}\left[{TMDSP}^{+\delta\:}\dots\:..{}^{-\delta\:}C{H}_{n}C{l}_{4-n}\right]$$

V $$\:\left[{TMDSP}^{+\delta\:}\dots\:..{}^{-\delta\:}C{H}_{n}C{l}_{4-n}\right]\to\:{TMDSP}^{+\delta\:}{Cl}^{-}+{CH}_{n}{Cl}_{3-n}\:contact\:ion\:pair$$

BTMSP in CHCl_3_ is unstable, decomposing in 40 s without exposure to radiation and forming a red colour. This may be attributed to the formation of an unstable CT-complex with CHCl_3_.

#### Laser activity of BTMSP.

The BTMSP dye demonstrates a significant Stokes shift from 3469 to 5335 cm^−1^ in various solvent environments. It also exhibits a high quantum fluorescence yield and an elevated extinction coefficient. As the concentration of BTMSP dye is increased up to 5 × 10^−4^ mol dm^−3^, there is no change in the emission spectra, indicating that BTMSP maintains its molecular integrity, without forming aggregates, in both its ground and excited states. Because of its favourable characteristics, BTMSP appears to be a promising dye laser material. BTMSP (5 × 10^−4^ mol dm^−3^) was stimulated in DMSO, DMF, CH_3_CN, and dioxane using a nitrogen pulsed laser with an excitation wavelength of 337.1 nm. The laser light produced has a pulse duration of 800 ps and a pulse energy of 1.48 mJ. The BTMSP solution was introduced into cuvettes with a path length of 10 mm, designed explicitly for oscillation and amplification. The wavelength-dependent output energy of the laser dye was determined to ascertain the lasing range in various solvents. The determination of the maximal gain coefficient (α) involved meticulously measuring the laser emission intensity (I_L_) across the entire cell length (L) and the intensity (I_L/2_) from half of the cell’s length. The calculation of laser gain emission is performed by the pertinent Eq. (15)^66^.

15 $$\:\alpha\:\left(\lambda\:\right)=\frac{2}{L}ln\left[\frac{{I}_{L}}{{I}_{\raisebox{1ex}{$L$}\!\left/\:\!\raisebox{-1ex}{$2$}\right.}}-1\right]$$

It is widely recognized that a net increase in photon emission is observed when the stimulated emission rate surpasses the combined effects of reabsorption and scattering-induced losses. Hence, the gain refers to the augmentation in the quantity of released photons, which is contingent upon both the wavelength and the intensity of the incoming light. The dye’s cross-sectional area for stimulated laser emission (σe) was evaluated at the laser’s emission peak, utilizing Eq. (16)^12^.

16 $$\:{\sigma\:}_{e}=\frac{{\lambda\:}^{4}F\left(\lambda\:\right)d\lambda\:}{8\pi\:c{n}^{2}}\:x\frac{{\varnothing\:}_{f}}{{\tau\:}_{f}}$$

In this context, λ represents the maximum fluorescence wavelength, n denotes the solvent refractive index, c refers to the speed of light, and F(λ) represents the normalised fluorescence intensity, where ∫ F(λ)d λ = 1. The value of σe closely approximates the value of high-quality laser dyes, such as rhodamine 6G and (POPOP) lasing dye^67^. The laser parameters and fluorescence lifetime (τ_f_) of BTMSP are listed in Table 2.

**Table 2:** Laser parameters of BTMSP in some Solvents.

Solvent	_L (max)_ (nm)ג	Lasing range (nm)	τ_f_ _(ns)_	^σ^_e_ (cm)^2^	α (Cm^−1^)
CH_3_CN DMSO DMF Dioxane	502 497 496 480	478–525 475–518 476–522 462–508	1.65 1.52 1.48 1.58	2.40 × 10^−16^ 2.60 × 10^−16^ 2.40 × 10^−16^ 2.25 × 10^−16^	1.23 1.20 0.96 1.26

#### Computational model

Structural optimization, electronic, and optical property analysis were conducted using Density Functional Theory (DFT) calculations^68–75^ using Gaussian 16[76,77]. The chosen functional for these calculations was the long-range-corrected M06-2X, known for its enhanced accuracy in carbon-based compounds^78–80^. The 6-311G++(d, p) basis set^69,81,82^ providing acceptable accuracy, was employed. For optical calculations, time-dependent DFT calculations were performed, considering the first six excited states. Parameters characterizing the nature of these excited states, such as the overlap between electron and hole density distributions (Sr index), were determined using Multiwfn software^83^. The Sr index is defined as $SS_{r} = \smallint S_{r} \left( r \right)dr = ~\smallint \sqrt {\rho ^{{hole}} \left( r \right)\rho ^{{ele}} \left( r \right)dr}$^84^, where *ρ* ^hole^(r) and *ρ* ^ele^(r) represent the density of hole and electron particles at a particular location, “r.“. To gauge the total charge transfer (CT) length, the D index^84^ was introduced: . Here, the magnitude of the CT length in the X/Y/Z directions is denoted by DX, DY, and DZ. Furthermore, H assesses the average degree of spatial extension of the hole and electron distribution in the X/Y/Z direction, HCT in the CT direction, and the H index offers an overall assessment, calculated as^84^. The |σ_hole_| and |σ_ele_| indices, referred to as σ_hole_ and σ_ele_ indices, respectively, quantify the overall Root Mean Square Deviation (RMSD) of hole and electron distributions. The t index^84^ measures the separation degree of hole and electron in the CT direction: . In the context of measuring charge-transfer length during elecron excitation, the Δr index was introduced as proposed by Thory^85^. It can be expressed as , where the indices i and a run over all occupied and virtual Molecular Orbitals (MOs), and ϕ is an orbital wave function. Various parameters, including excitation energy (E_opt_), emission energy (E_Emss_), Sr index, oscillator strength, difference in RMSD index, radiative lifetime, and emission (Emss.) and absorption (Abs.) transition dipole moment in different solvents, were obtained using M06-2X/6-31G++(d, p) based on electronic absorption and emission calculations, facilitated by the Multiwfn software^83^.

#### Optimized and stability investigation

In Fig. S5a, the optimized molecular structures (MS) of BTMSP are seen in a gaseous condition, and Table S2 furnishes crucial parameters for the optimized MS, which involves determining the bond lengths in angstroms (Å), bond angles, and dihedral angles (measured in degrees) of the BTMSP molecule. The following observations can be made from these results: (i) The dihedral angles suggest a planar conformation of the BTMSP MS, wherein the vinyl (C = C) and methoxy (-OCH_3_) groups lie in the same plane as BTMSP. (ii) The C9-C10 bond length is shorter than the C2-C9 and C2-C3 bond lengths in the BTMSP MS due to an increased bond order. (iii) The bond angle values indicate SP^2^ hybridization of the atoms in the BTMSP MS. To evaluate the stability of the BTMSP MS, we computed the binding energy (BE) and conducted frequency calculations. The BE was calculated using the formula: BE = (N_C_E_C_ + N_H_E_H_ + N_N_E_N_ + N_O_E_O_ - E_t_)/N_t_, where N_C_, N_H_, N_N_, N_O_, and N_t_ denote the numbers of C, H, N, O, and the total number of atoms, respectively. E_C_, E_H_, E_N_, E_O_, and E_t_ represent the total energies of the C, H, N, O, and the final compound, respectively. The calculated BE of 5.812 eV, as depicted in Fig. S5b, signifies the BTMSP MS. To further evaluate the structural stability, we analyzed the infrared (IR) spectra derived from frequency calculations. The presence of actual vibrational frequencies, as depicted in Fig. S4b, affirms that there are no saddle points on the potential energy surface, offering additional proof of the dynamic stability of the examined structure.

#### HOMO and LUMO investigation

**Fig. 7:**
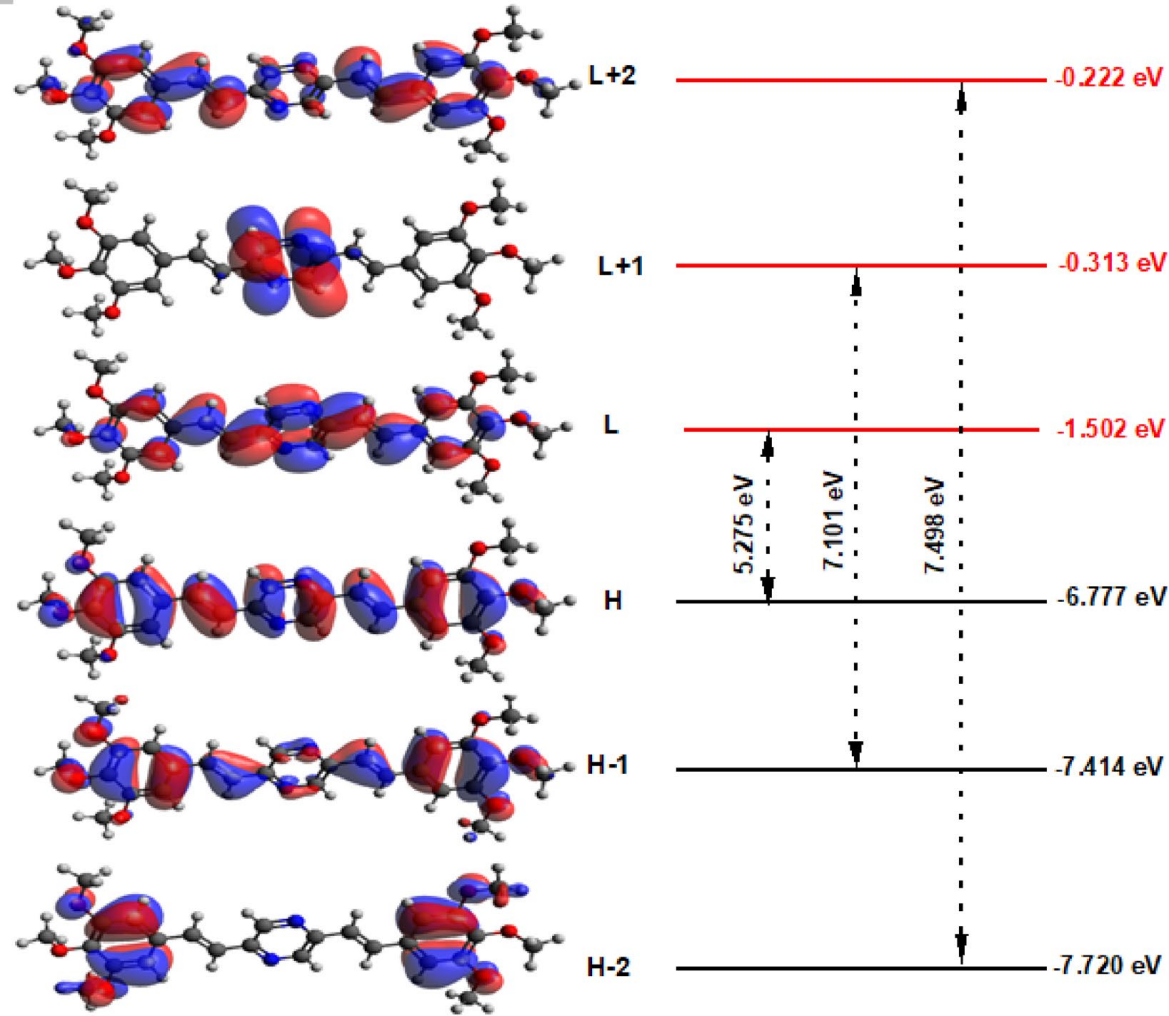
Graphical presentation of HOMO-2 (H-2), HOMO-1(H-1), HOMO (H), LUMO-2 (L-2), LUMO-1 (L-1), LUMO (L) molecular orbitals (MOs) and energy gaps (E_g_) for BTMSP MS.

Understanding molecular orbitals’ composition and energy levels (MOs) is fundamental for gaining insights into the electronic behavior of molecular systems^86^. In this context, E_HOMO_ energy signifies the molecule’s ability to donate electrons, while E_LUMO_ energy characterizes its capacity to accept electrons^86^. Moreover, the energy gap (E_g_) between E_HOMO_ and E_LUMO_ is a crucial factor influencing molecular chemical stability and electron conductivity, which is essential for molecular electrical transport^86^. Figure 7 presents graphical representations of various MOs (HOMO-2, HOMO-1, HOMO, LUMO-2, LUMO-1, and LUMO) for the BTMSP MS. Across the entire molecular backbone of BTMSP, the electron density (ED) in the HOMO (H) and LUMO (L), as well as HOMO-1 (H-1) and LUMO + 2 (L + 2) MOs, is distributed over the entire molecule, except for above and below the methoxy groups, where there are small contributions. In contrast, the ED in HOMO-2 (H-2) and LUMO + 1 (L + 1) MOs is localized to the terminal phenyl groups and the pyridine rings, respectively, as depicted in Fig. 7. The energy levels of these MOs, denoted as E_HOMO-2_ (E_H-2_), E_HOMO-1_ (E_H-1_), E_HOMO_ (E_H_), E_LUMO-2_ (E_L-2_), E_LUMO-1_ (E_L-1_), and E_LUMO_ (E_L_), as well as the energy gaps between them, such as H-2 and L-2 (E_g2_), H-1 and L-1 (E_g1_), and H and L (E_g_), in the gaseous state for BTMSP MS have been calculated. The results are presented in Fig. S4. Specifically, the calculated values for E_H_ and E_L_ for the studied BTMSP MS are − 6.77 and − 1.502 eV, respectively, as shown in Fig. 7. Using these values, the energy gap (E_g_) is determined to be 5.275 eV. When comparing these results with previously reported findings from the literature^39,70^it is evident that the E_g_ value for the studied BTMSP MS is larger than those reported in the prior studies^39,70^. This difference in the energy gap could have significant implications for the molecular electronic properties and stability of BTMSP, which merits further investigation and consideration in relevant applications.

#### ESP and DOS investigations

**Fig. 8:**
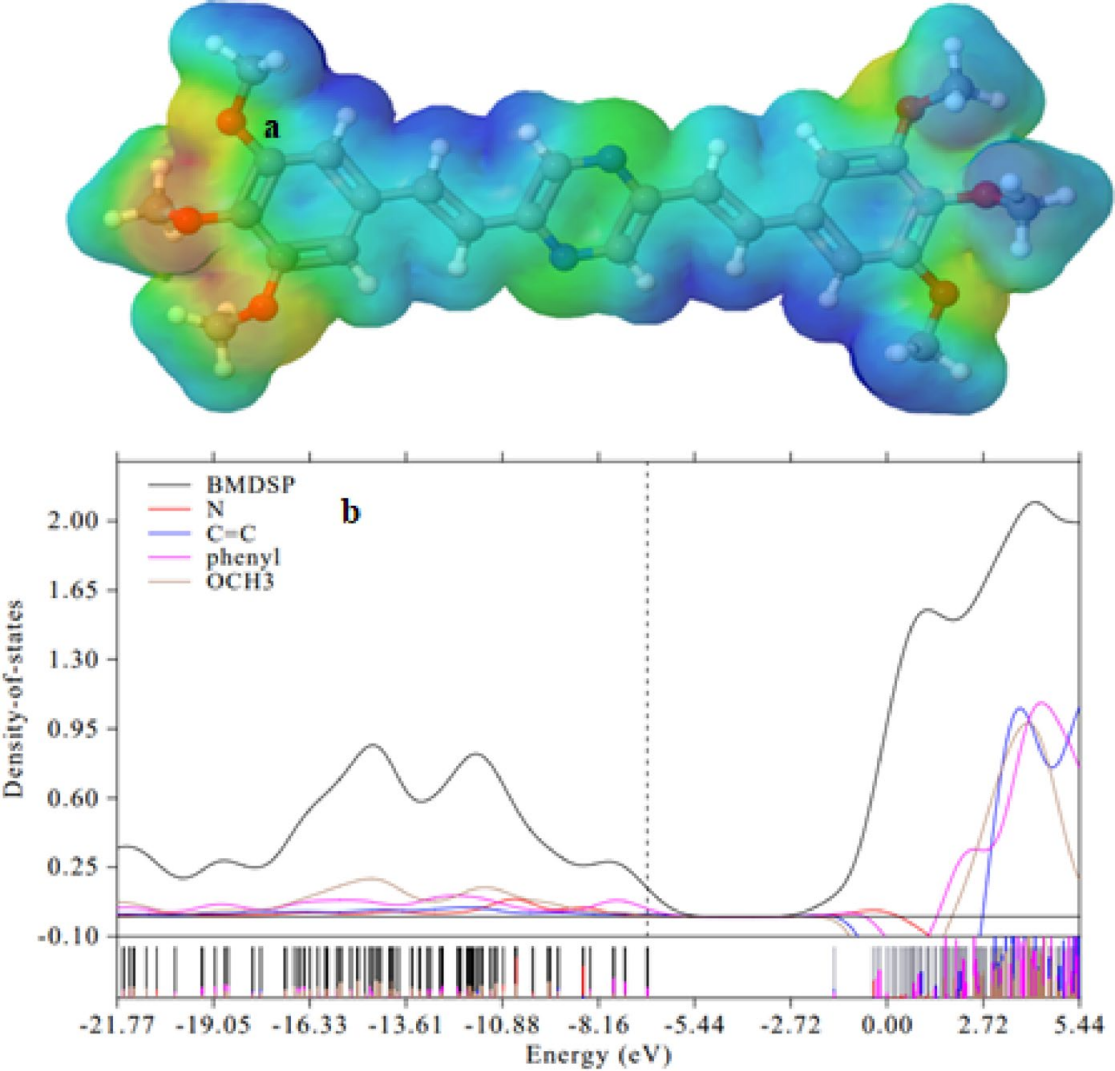
Electrostatic potential map (ESP) (**a**), total and partial density of states of BTMSP (**b**) in a gaseous state.

An electrostatic potential (ESP) study was performed to identify the electrophilic and nucleophilic regions in the BTMSP compound^87^. Figure 8a displays the ESP, with a color scheme representing different charge densities. The color scheme is as follows: red and yellow for regions with an electron-rich, partially negative charge; blue for regions with an electron-deficient, partially positive charge; light blue for slightly electron-deficient regions; yellow for slightly electron-rich regions, and green for neutral (zero potential) regions (refer to Fig. 8a). The nitrogen and oxygen atoms are surrounded by a negative region (red and yellow colors) in the ESP surface, indicating that this area is electron-rich and susceptible to electrophilic attacks. On the other hand, the hydrogen atoms linked to carbon atoms (C-H) exhibit a positive region (blue color) in the ESP, suggesting that these sites are electron-deficient and prone to nucleophilic attacks. Therefore, based on the ESP analysis, N and O atoms represent an electrophilic site, while the C-H bonds serve as nucleophilic sites in the BTMSP compound. This information is valuable for predicting potential reaction sites and understanding the reactivity of these compounds.

The PDOS (Partial Density of States) and TDOS (Total Density of States) presented in Fig. 8b have been derived through a detailed analysis of the Gaussian output file using the Multiwfn software, which calculates the percentage contribution of each atom and group to the molecular orbitals. These results provide valuable insights into the electronic structure of the studied compound. One striking observation from the PDOS spectra is the overlap of peaks, with the OCH_3_ peaks appearing higher than those of nitrogen, C = C, and phenyl groups in the PDOS of the molecule, as shown in Fig. 8b. This observation is consistent with the results obtained from ESP calculations and can be attributed to several factors related to the electronic structure and bonding properties of the molecule. Here are a few possible explanations for these results: (i) Hybridization and Molecular Orbitals: The overlapping PDOS spectra suggest the presence of strong orbital interactions and electron delocalization within the molecule. This could indicate the formation of molecular orbitals that involve multiple atoms, leading to shared electron densities. (ii) Conjugation and Resonance: The molecular structure of BTMSP contains conjugated systems and resonance structures, which allow for the delocalization of electrons across the molecule. This can contribute to the overlapping PDOS spectra and potentially result in enhanced electron densities on the carbon atoms. The higher PDOS peaks observed for OCH_3_ compared to nitrogen, phenyl, and C = C groups might be attributed to the orbital energies of these groups. The PDOS spectra in Fig. 8b reveal interesting details about the electronic structure of the molecule. In the HOMO, multiple DOS peaks are associated with OCH_3_, nitrogen, phenyl, and C = C groups. This suggests that in the HOMO, where electrons are primarily localized, these different groups contribute to multiple DOS peaks due to their distinct electronic configurations and interactions with neighboring atoms. However, in the LUMO, which represents the lowest energy level available for electron acceptance, there is only a single peak for the OCH_3_, phenyl, and C = C groups. This could be indicative of carbon atoms having the highest affinity for accepting electrons among the atoms in the molecule in the LUMO. The absence of DOS peaks for nitrogen atoms in the LUMO suggests that nitrogen has a lower affinity for accepting electrons compared to carbon. Overall, the PDOS analysis provides valuable information about the electronic properties and interactions within the studied molecule, shedding light on its electronic structure and bonding characteristics.

#### Calculated absorption spectra

In our investigation of the BTMSP compound, we conducted a comprehensive study of its UV-Vis absorption and emission spectra in three distinct solvents: ethanol, acetonitrile, and DMSO (Fig. S6). Our goal was to precisely compute these spectra and compare them with experimental data, aiming to pinpoint the most appropriate functional and basis set for accurate results. Our selected computational approach involved employing the TD/M06-2X/6-311G++(d, p) method, which is known for its efficacy when diffuse Functions are integrated, ensuring that we achieved highly accurate UV-Vis absorption and emission spectra compared to the experimental data. The experimental UV-Vis absorption spectra revealed absorption peaks ranging from 407 to 418 nm across all three solvents, as outlined in Table S3. We also obtained the maximum emission spectra in these solvents, which we have documented in Table S3. These distinctive absorption features indicate π-π* electronic transitions, a critical aspect of our investigation. Our computational calculations generated electronic absorption and emission spectra for BTMSP in ethanol, acetonitrile, and DMSO. These spectra were found to exhibit single electronic transitions for each solvent. Specifically, the computed electronic absorption peaks for BTMSP were observed at 421.67 nm in ethanol, 422.93 nm in acetonitrile, and 421.23 nm in DMSO, each corresponding to the transition from the HOMO to the LuMO. Furthermore, the computed electronic emission spectra for BTMSP in these solvents displayed peaks at 576.49 nm in ethanol, 578.6 nm in acetonitrile, and 579.76 nm in DMSO. These emission peaks signify the relaxation of excited states back to the ground state, providing essential insights into the molecule’s behavior. Upon thorough comparison of the experimental and computational results, as meticulously documented in Table S3, it is evident that our computational approach closely aligns with the experimental findings. The minor variations observed between the two sets of data were well within reasonable limits, with percentage errors ranging from approximately − 0.77% to −3.89% for the absorption spectra and − 13.82% to −18.63% for the emission spectra. These discrepancies can be attributed to factors such as solvent-solute interactions and the precision of our measurement instruments. Nevertheless, overall, our study demonstrates a commendable agreement between the computational and experimental datasets, affirming the reliability of the TD/M06-2X/6-311G++(d, p) method, especially when diffuse functions are included, in accurately elucidating the electronic properties of BTMSP in the specified solvents.

#### Characterization of excited states

In our exploration of the excited states of the BTMSP molecule, we employed a suite of indices to thoroughly characterize these states, providing valuable insights into their electronic structure and properties. The indices utilized, namely S_r_, D, t, E_c_, and Δr, contribute to understanding the overlap between electron and hole densities, the centroid coordinates of holes and electrons, the degree of separation between these charges, the hole-electron Coulomb attractive energy, and the charge-transfer length, respectively. In particular, our focus was on six representative excitation states, denoted as S_1_, S_2_, S_3_, S_4_, S_5_, and S_6_. The Δr index emerged as a pivotal parameter in our analysis, quantifying the charge-transfer length during electron excitation. The application of the Δr index, typically with a threshold set at 2.0 Å, provided a criterion for discerning and characterizing the nature of charge transfer in the excited states of the BTMSP molecule^85,88^the excitations from the ground state (S_0_) to the 1 st, 2nd, 3rd, 4th, 5th, and 6th excited states were primarily classified as local excitations (LE) due to their relatively modest Δr indices. These findings align with previous research emphasizing the use of the Δr index to distinguish between LE and charge transfer excitations (CT)^85,88^. Moving on to the D index, our analysis revealed that the centroids of the blue (hole) and green (electron) isosurfaces, representing the centroids of C_hole_ and C_ele_, were closely positioned for all excitations. This proximity strongly suggests that these excitations should be classified as local excitations (LE). The Sr index, which provides insights into the degree of spatial overlap between hole and electron distributions, demonstrated that most excited states exhibited relatively large Sr indices, except for S_0_→S_3_ and S_0_→S_6_, where the Sr values were lower than 0.50. These exceptions can be attributed to highly localized π-π* type excitations occurring predominantly in the middle pyridine ring. In contrast, S_0_→S_1_, S_0_→S_2_, S_0_→S_4_, and S_0_→S_5_ displayed notably large Sr values due to their highly localized π-π* type excitations occurring on the benzene rings, vinyl, and pyridine rings.

**Fig. 9:**
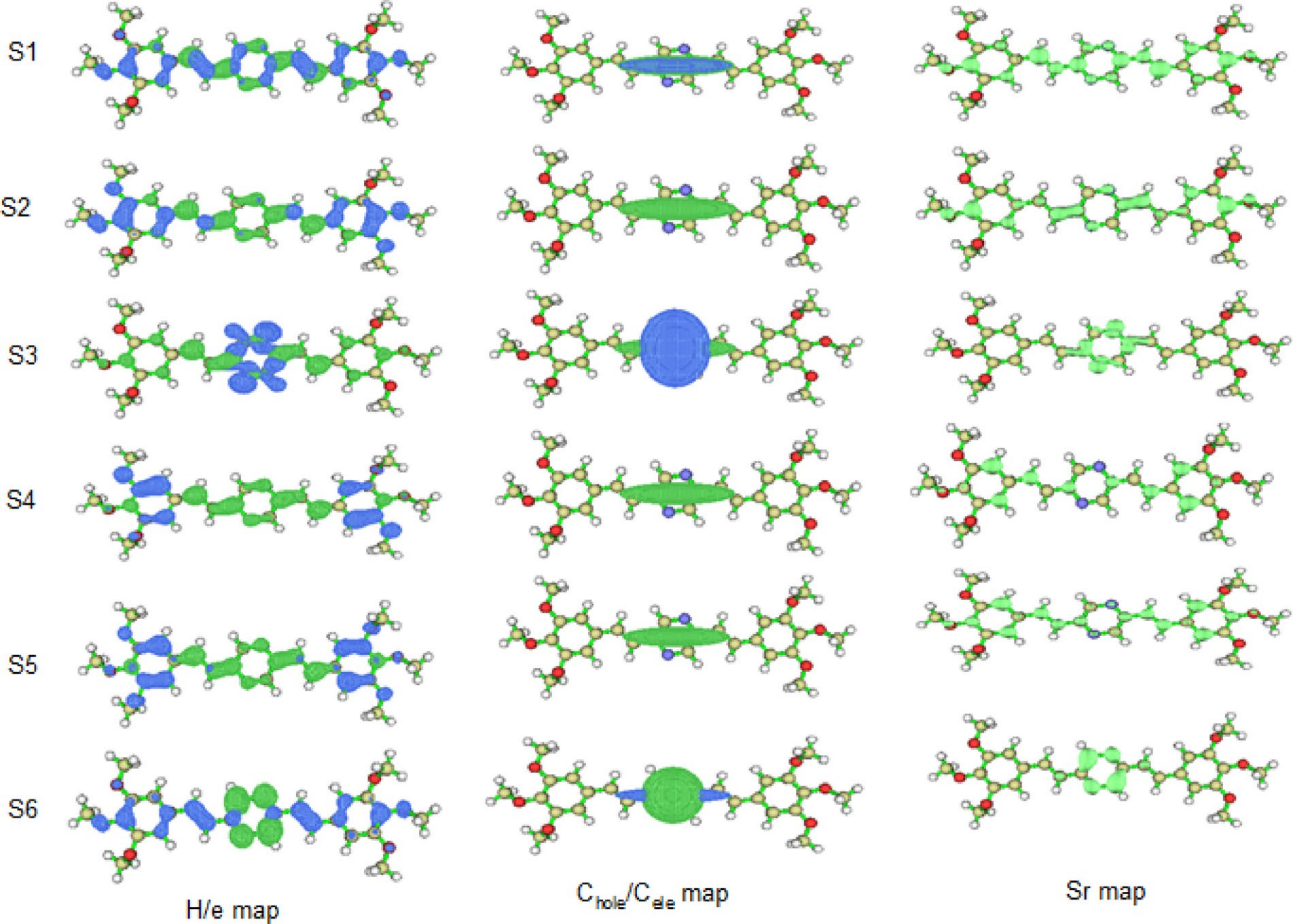
Hole/electron maps of BTMSP MS.

The H index, which reflects the breadth of the average distribution of hole and electron densities, highlighted distinct patterns. The H indices were notably large for S_0_→S_2_, S_0_→S_4_, and S_0_→S_5_, where the hole predominantly resided on the right and left benzene rings while electrons were distributed across the vinyl and pyridine rings. In contrast, S_0_→S_1_ displayed a relatively equal distribution of holes and electrons, resulting in a lower H index compared to S_0_→S_2_, S_0_→S_4_, and S_0_→S_5_. Finally, S_0_→S_3_ and S_0_→S_6_ exhibited lower H index values than other excitations due to their unique distributions of holes and electrons. The t indices for all excitations were negative, signifying a minimal degree of separation between holes and electrons. Consequently, it is more appropriate to classify S_0_→S_1_, S_2_, S_3_, S_4_, S_5_, and S_6_ as local excitations (LE). Table S4 further provided insights into the hole-electron Coulomb attractive energy, closely related to the electron excitation characteristics. Notably, the D index played a pivotal role in this context. Larger D indices indicated greater separation between the main distribution regions of hole and electron densities, resulting in weaker Coulomb attractive energy. As such, for excitations like S_0_→S_1_/S_2_/S_3_/S_4_/S_5_/S_6_ Fig. 9, where the D indices were minimal, and the H index indicated narrow spatial extent for holes and electrons, we inferred that the corresponding Coulomb attraction was powerful. In conclusion, our thorough characterization of excited states through these indices provides valuable insights into the electronic structure and properties of the BTMSP molecule. These findings are instrumental in understanding the nature of electronic transitions and charge distributions within the molecule, enriching our comprehension of its behavior and reactivity.

#### Solvent Polarity effects on optical parameters

In our comprehensive study, we explored various optical parameters to assess the impact of solvent polarity on the behavior of the BTMSP molecule, with the results meticulously compiled in Table 3. Our primary objective was to investigate how different solvents, characterized by varying degrees of polarity, influenced these parameters. Specifically, the solvents in question were arranged in ascending polarity: Acetonitrile < Ethanol < DMSO. The excitation energy (E_opt_) and emission energy (E_Emss_) values, which were recorded in Table 3, represent the global absorption and emission maxima for most of the systems under investigation. Notably, the excitations associated with E_opt_ correspond to the absorption spectra’ peak, while the relaxations marked by E_Emss_ represent the global emission maxima. Upon examining the values of E_opt_ and E_Emss_ in Table 3, it becomes apparent that the absorption and emission spectra of BTMSP in DMSO exhibit a noticeable redshift compared to ethanol and acetonitrile. This phenomenon can be attributed to DMSO’s considerably higher polarity than ethanol and acetonitrile. This redshift reflects the influence of solvent polarity on the electronic transitions within the molecule. We further explored the concept of the Stokes shift, defined as the energy difference between E_opt_ (absorption) and E_Emss_ (emission). This shift serves as a valuable indicator of the extent of nonradiative relaxation, which involves changes in the geometric structure of BTMSP. The degree of geometric change is quantified by the Root Mean Square Deviation (RMSD) values listed in Table 3. Notably, RMSD is calculated concerning the optimized ground state structure. We observed a general reduction in Stokes shift as solvent polarity increased, especially in ethanol and DMSO. A minor Stokes shift signifies a lesser energy change during nonradiative relaxation, suggesting that associated geometric changes are relatively small. Furthermore, the lower Sr indices observed in polar solvents, particularly DMSO, reflect the influence of solvent polarity. Due to DMSO’s significantly higher polarity compared to ethanol and acetonitrile, the oscillator strengths of absorption and emission spectra for BTMSP in DMSO were found to be higher than those in ethanol and acetonitrile.

Table 3 offers valuable information about the transition dipole moments observed in the fluorescence and absorption spectra. Interestingly, the transition dipole moment in the fluorescence spectra was determined to exceed that observed in the absorption spectra. Moreover, BTMSP exhibited lower transition dipole moments in polar solvents such as ethanol and DMSO than in acetonitrile, underscoring the impact of solvent polarity on this parameter. To delve into excited state lifetimes (τ), we employed the formula τ = 1.499/*f* E2)^68^, where E represents the excitation energy of different electronic states in cm^[- 1^, and f denotes the oscillator strength of the electronic state. The calculated τ values in Table 3 reveal that τ in DMSO is greater than that in ethanol and acetonitrile. This discrepancy can be attributed to DMSO’s high polarity relative to ethanol and acetonitrile solvents, which has a pronounced effect on excited-state lifetimes.

In summary, our study delves into a range of optical parameters to elucidate the influence of solvent polarity on the behavior of BTMSP. These findings offer valuable insights into the molecule’s electronic transitions, geometric changes, and excited-state lifetimes, all contributing to our understanding of its behavior in various solvent environments.

**Table 3:** Excitation energy (E_opt_), emission energy (E_Emss_), Sr index, oscillator strength, difference in RMSD index, radiative lifetime and emission (Emss.) and absorption (Abs.) transition dipole moment in various solvents.

Solvent	E_opt_ (eV)	E_emss_ (eV)	Stoke shift (eV)	Radiative lifetime (ns)	Sr (a.u.)	RMSD (A)	Transition dipole moment (au)		Oscillator strength	
							Emss.	Abs.	Ems.	Abs.
Ethanol	2.940	2.150	0.789	1.073	0.80313	−1.097	7.263	5.485	2.483	1.921
Acetonitrile	2.943	2.142	0.801	1.069	0.80306	−1.095	7.287	5.486	2.486	1.917
DMSO	2.931	2.138	0.793	1.077	0.80306	−1.096	7.269	5.485	2.488	1.933

#### Fluorescence quenching of BTMSP dye using silver nanoparticles

**Fig. 10:**
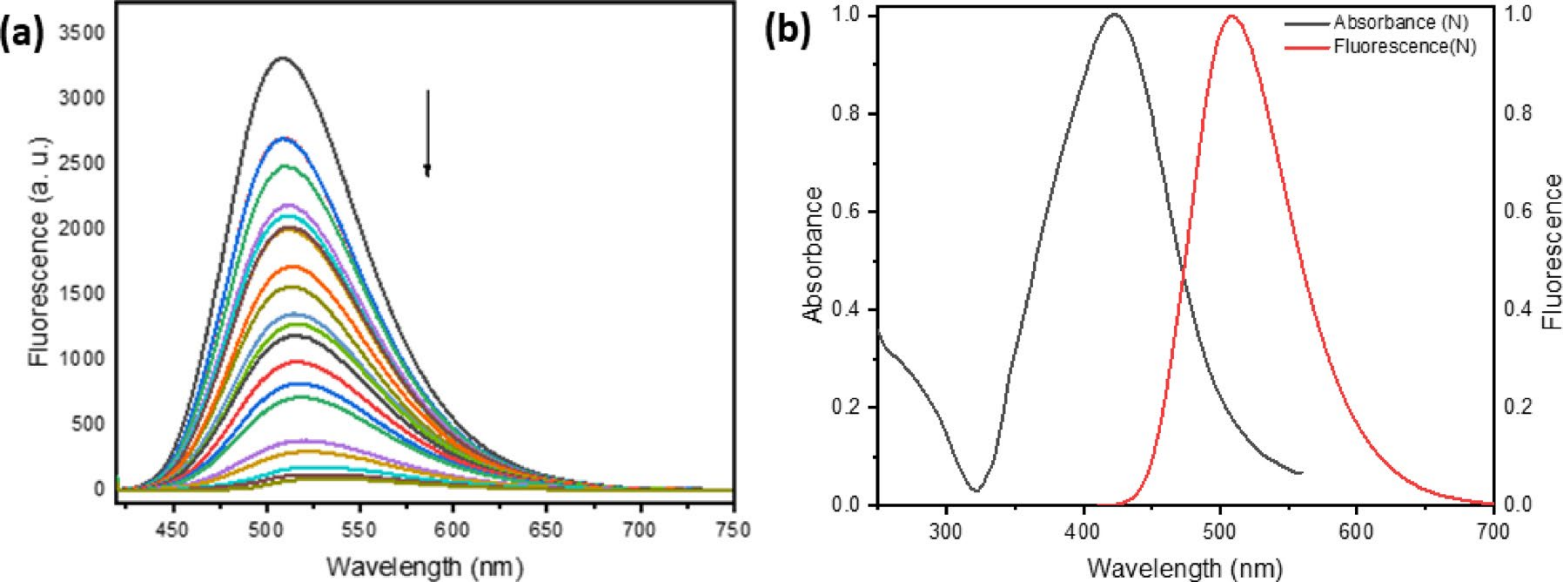
Fluorescence quenching of 1 × 10^−5^ mol dm^−3^ of dye in ethanol at different concentrations of AgNPs, 0.04, 0.14, 0.24, 0.34, 0.44, 0.53, 0.63, 0.73, 0.83, 0.93, 1.02, 1.12, 1.22, 1.32, 1.42, 1.51, 1.61, 1.71, 1.81, 1.91 × 10^−11^ mol dm^−3^, (λ_ex_ = 410 nm) (a**a** and Absorption spectra of AgNPs and Normalized fluorescence spectra of BTMSP λ_ex_ = 400 nm (**b**).

One way to study fluorophores’ microenvironment and molecular interactions in a solution is by doing fluorescence quenching experiments. Various quantities of silver nanoparticles (AgNPs) in ethanol were tested at room temperature to determine the fluorescence emission of BTMSP. The fluorescence emission spectrum of a solution containing BTMSP at a concentration of 1 × 10^−5^ mol dm^−3^ in ethanol reveals a peak intensity at 508 nm when excited at a wavelength of 410 nm. As depicted in Fig. 10a, the fluorescence intensity experiences a significant decrease while the position of dye fluorescence bands stays unaltered, even with the rise in the quencher (AgNPs) concentration. This observation suggests that there is no presence of an emissive exciplex under the experimental conditions being considered. The presence of 10% citrate as a capping agent did not result in any observed quenching of the examined dye, suggesting that the fluorescence quenching is solely attributed to the AgNPs. Upon adding AgNPs to the dye solution, the spectral pattern and absorption maxima of the dye solution were unaltered, indicating no ground-state interaction between BTMSP and AgNPs. To ascertain the Stern-Volmer constant (Ksv) for BTMSP employing AgNPs as a quencher, the fluorescence quenching behaviour might be evaluated utilising the Stern-Volmer Eq. (17)^89^:

17 $$\:\frac{{I}_{0}}{I}=1+{K}_{SV}\left[Ag\right]$$

**Fig. 11:**
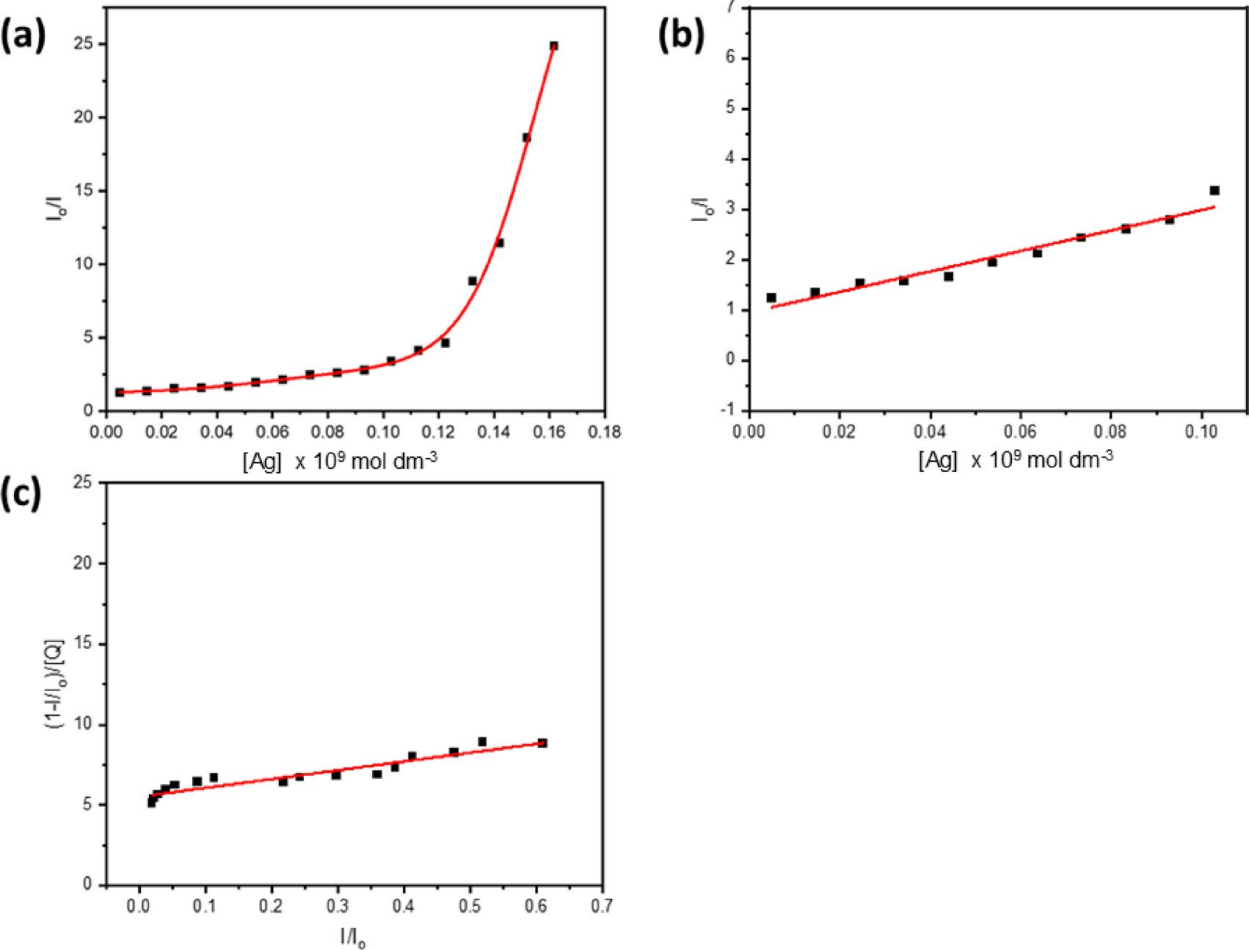
Stern–Volmer relationship for fluorescence quenching of BTMSP by AgNPs in EtOH (**a**). linear Stern -Volmer plot of TMDST at lower concentration of AgNPs (**b**) and [1 − (I/I_0_)]/[Q] versus (I/I_0_) BTMSP dye (**c**).

The fluorescence intensities in the presence and absence of the quencher concentration [Ag] are denoted as I_o_ and I, respectively. As illustrated in Fig. 11a, the Stern-Volmer plot for BTMSP was found to be nonlinear, with a positive deviation at larger silver nanoparticle concentrations. Similar experimental results were reported by others^90–94^. Positive divergence from linearity: This indicates that the quenching is due to both dynamic and static quenching types co-occurring, as opposed to being entirely dynamic. A straight line was obtained at a moderately low concentration of silver nanoparticles (0.0049–0.12 × 10^−9^ mol dm^−3^) as shown in Fig. 11b. Stern-Volmer constant Ksv was determined as 2.3 × 10^10^ mol^−1^ dm^3^. The bimolecular quenching rate constant k_q_ (= K_SV_/τ) was determined as 1.5 × 10^18^ mol^−1^ dm^3^ s^−1^ (τ of BTMSP in EtOH = 1.45 ns). The exceptionally elevated rate constants for fluorescence quenching are ascribed to the phenomenon known as hyper- or superfluorescence quenching^30^. Multiple explanations exist for the observed behaviour of metallic nanoparticles’ super-quenching fluorescence. To begin with, the metallic nanoparticles’ surface plasmon resonance (SPR) absorption band has a high absorption coefficient. Secondly, the spherical form of AgNPs allows for energy transfer to occur regardless of the donor molecule’s orientation concerning the nanometal surface. Finally, as shown in Fig. 10b, the super-quenching effect is further amplified by the donor molecule’s emission overlapping with the SPR absorption band.

##### Fluorescence quenching by AgNPs

The two primary causes of the positive deviations shown in Stern-Volmer (S-V) plots can be explained. They first originate from static quenching, which is ascribed to the ground-state complexes that form between the quencher and fluorophore molecules. Furthermore, the presence of the quencher molecule within the quenching field of action causes a temporary component of static quenching. Models that consider the creation of ground-state complexes and the quenching sphere of action explain these events. The absence of any alteration in the electronic absorption spectrum of AgNPs for BTMSP leads to the rejection of the deviation attributed to ground-state complex formation. For this study, we used the static quenching model with a sphere of action according to Eq. 29 and a linear connection depicted in Fig. 11c with correlation coefficients 0.97. The least-squares fit was used to determine K_SV_ and V from the slope and intercept. Ksv for dynamic quenching of BTMSP was determined to be 5.47 × 10^9^ mol^−1^ dm^3^, while V for static quenching was calculated to be 5.5 × 10^9^ mol^−1^ dm^3^. The rate constant for bimolecular quenching (k_q_= K_SV_/τ) is 3.7 × 10^18^ mol^−1^ dm^3^ s^−1^. The larger k_q_ values indicate more effective fluorescence quenching. The static quenching constant (V) is close to K_SV_ in magnitude, which explains the greater departure from the linear trend shown in the S-V graphs in Fig. 11a.

18 $$\:\frac{\left[1-\left(I/{I}_{0}\right)\right]}{\left[Q\right]}={K}_{SV}\left(\frac{I}{{I}_{0}}\right)+V$$

**Fig. 12:**
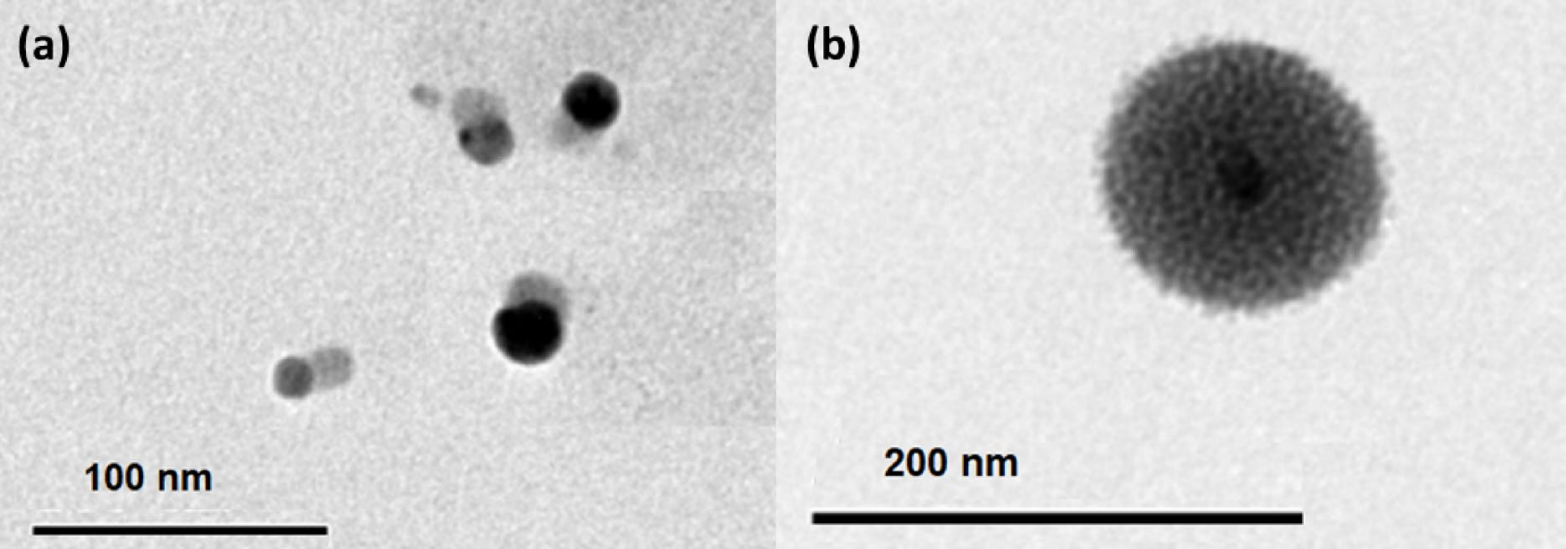
TEM image of AgNPs (**a**), TEM image of aggregated dye around AgNPs (**b**).

Using Eq. 30, we determined that the radius of the active sphere volume of static quenching, denoted by the symbol (r), is 1.28 × 10^−4^ centimetres. Both static and dynamic effects are supported by the magnitudes of the static quenching constant V (V = 5.5 × 10^9^ mol^−1^ dm^3^) and the radius r of the sphere of action (kinetic distance). In view of these considerations, it seems most appropriate to examine the static quenching phenomenon in terms of sphere action. The radii of the fluorophore (R_Y_) and the quencher (R_Q_) molecules were determined as R_Y_ = 2.1 Ă and R_Q_ = 150 Ă according to Edward^95^. The encounter distance (R) between BTMSP and AgNPs is determined by adding the molecular radii of R_Y_ and R_Q_, resulting in a value of 150.21Å. To assess whether the observed response aligns with the sphere of action model, this value is compared to the kinetic distance (r). It’s worth noting that the kinetic distance (r) is greater than the encounter distance (R). According to Andreet al.^96^, when the encounter distance (R) falls within the range of the kinetic distance r, the static effect is observed, particularly in steady-state experiments, regardless of the formation of ground-state complexes, assuming that the reactions are diffusion-limited. Since r is greater than the encounter distance R, it seems that the sphere of action approach can be used effectively. It’s also important to note that if static and dynamic quenching happen simultaneously, the Stern-Volmer plot will likely go positively. The observed positive deviations in the S-V plot can be ascribed to the pre-concentration of BTMSP molecules on the surface of nanoparticles, leading to the creation of micelle-like self-assembly, as depicted in Fig. 12a,b. This self-assembly process ultimately leads to significant fluorescence quenching.

19 $$\:V=\frac{4}{3}\pi\:{r}^{3}\left(\frac{N}{1000}\right)$$

Benesi and Hildebrand’s approach was used to determine the apparent association constant (K_ass_), which was then used to analyse further the interaction between adsorbed BTMSP molecules and AgNPs^97^ Eqs. (20–22).

20 $$\:TMDSP+Ag\to\:\left[TMDSP\dots\:\dots\:Ag\right]$$

21 $$\:{k}_{ass}=\frac{\left[TMDSP\dots\:.Ag\right]}{\left[TMDST\right]\left[Ag\right]}$$

22 $$\:\frac{1}{{F}^{o}-F}=\frac{1}{({F}^{o}-{F}^{'})}+\frac{1}{k}\:x\:\frac{1}{\left({F}^{o}-{F}^{'}\right)\left[Ag\right]}$$

**Fig. 13:**
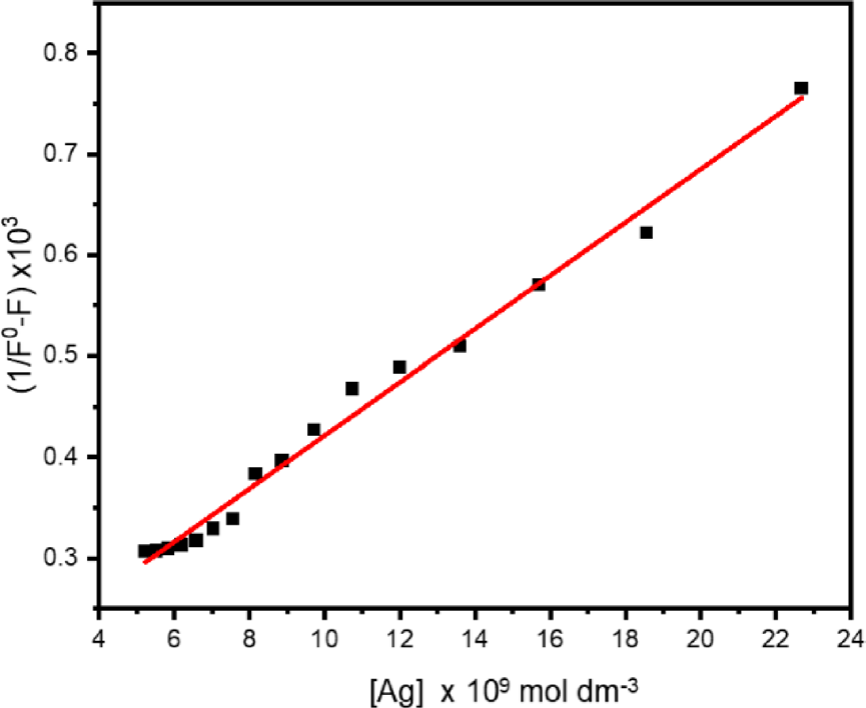
Interaction between BTMSP dye and AgNPs using the Benesi-Hildebrand.

In this context, K_ass_ represents the association constant, F^o^ denotes the fluorescence intensity of BTMSP in the absence of AgNPs, F’ represents the fluorescence intensity of dye molecules adsorbed onto silver (Ag), and F represents the maximum fluorescence intensity. The values of K_ass_ were determined using calculations based on the curve of $\:\frac{1}{{F}^{o}-F}$ vs 1/[Ag], as depicted in Fig. 13. The resulting values were determined to be 5.98 × 10^9^. The elevated value of K_ass_ signifies a robust correlation between the BTMSP and AgNPs.

#### The impact of micelle formation

**Fig. 14:**
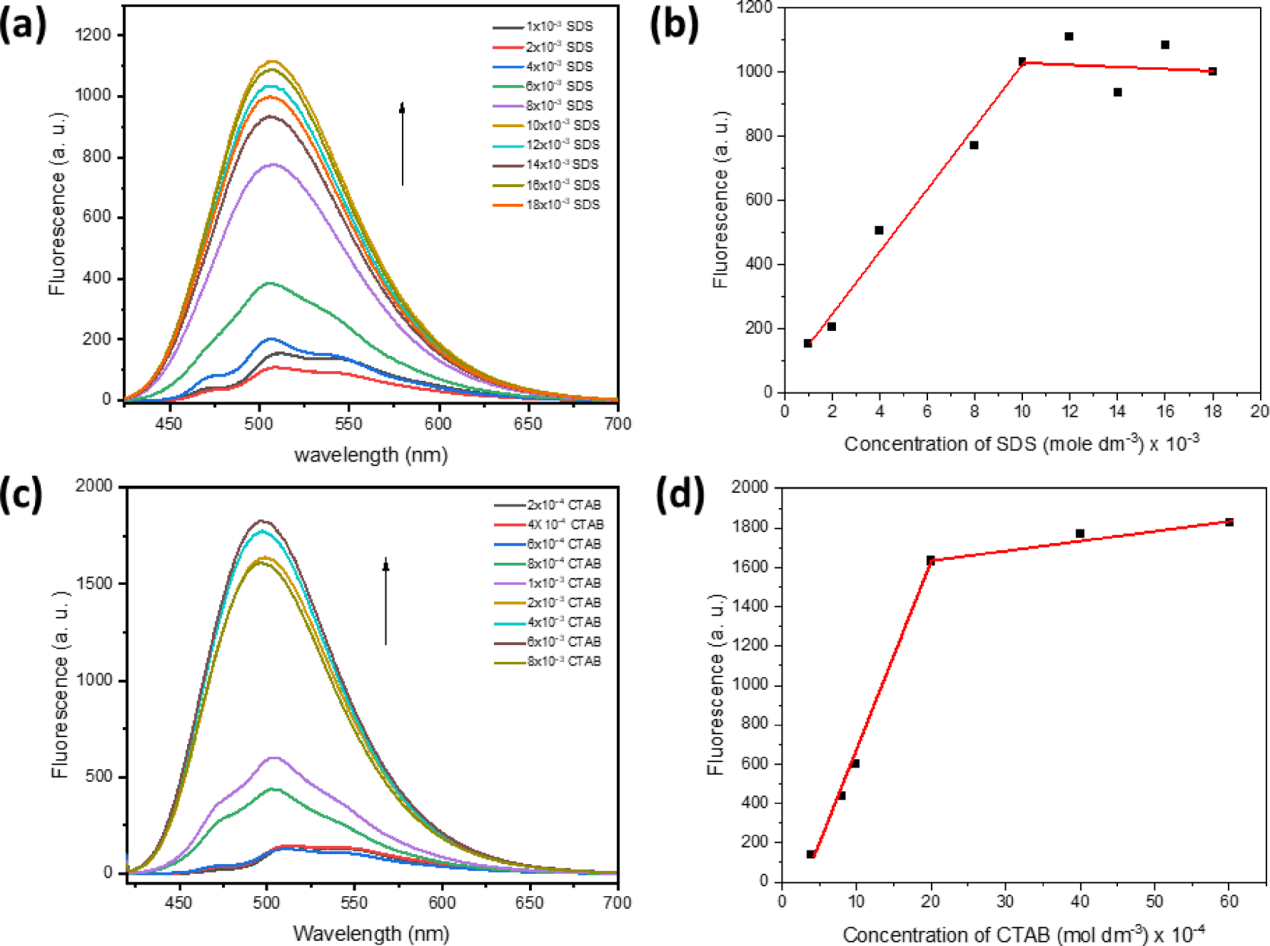
The fluorescence of BTMSP dye in the presence of SDS (**a**), the effect of SDS on fluorescence of the BTMSP dye (**b**). The fluorescence of BTMSP dye in the presence of CTAB (**c**), the effect of CTAB on the fluorescence of the investigated dye (**d**).

Fluorescence spectra were additionally recorded within a micellar environment, which included sodium dodecyl sulfate (SDS) and cetyltrimethyl ammonium bromide (CTAB). This was achieved using a solution containing 1 × 10^−5^ mol dm^−3^ of BTMSP dye, as depicted in Fig. 14a,c. A notable and rapid alteration in fluorescence intensity becomes evident at concentrations of 9.6 × 10^−3^ mol dm^−3^ for CTAB and 20 × 10^−4^ mol dm^−3^ for SDS, as shown in Fig. 14b,d. These concentrations are proximate to the critical micelle concentrations of these respective substances^98,99^. A further piece of evidence that the restricted hydrophobic core of the micelles used to dissolve BTMSP dye is the fact that the emission intensity is higher in the micellar environment than in water. The observed fluorescence quenching can be plausibly attributed to the impact of frictional forces and a reduction in the available solvent-free volume, constraining the free rotational motion of molecules and thus leading to the observed behaviour. This phenomenon is consistent with established principles in molecular dynamics and fluorescence spectroscopy^100^. Therefore, BTMSP dye is employed to determine various micelles’ critical micelle concentration (CMC).

## Conclusion

BTMSP dye was synthesized by aldol condensation, and the structure was investigated and confirmed by ^1^H and ^13^C NMR. The investigation focuses on examining the behavior of BTMSP dye in various solvents, highlighting its notable characteristics such as good photostability, high quantum yield of fluorescence, and high extinction coefficient. These exceptional attributes position BTMSP as a favorable choice for laser applications of dye. The laser emission range of BTMSP dye is 475–518 nm. Using solvatochromic techniques, such as the equations published by Lippert-Mataga, Bakhshiev, and Kawski-Chamma-Viallet, the difference in dipole moment of the first excited state with that of the ground state was calculated. Despite being less polar in its ground state, BTMSP dye has a dipole moment of 6.22 Debye in its excited singlet state, as opposed to 2.02 Debye in its ground state. The basicity of the dye increases when it is excited, as shown by a rise in the excited-state pKa value relative to the ground state. BTMSP dye exhibits limited solubility in aqueous solutions; however, it can be effectively solubilized in cationic and anionic micelles. Hence, the BTMSP can be employed for the identification of the CMC of SDS and CTAB. The fluorescence technique has also examined the interaction between BTMSP and colloidal AgNPs in ethanol. The Stern-Volmer plot of BTMSP exhibited a non-linear trend characterized by a positive deviation, suggesting that dynamic mechanisms are not the only ones that control the quenching process, but instead involve a combination of dynamic and static quenching phenomena. The phenomenon of fluorescence superquenching is widely recognized in scientific literature. It occurs when nanoparticles are used as quenchers, which can efficiently quench fluorescence even at very low concentrations. In our investigation of fluorescence quenching, we utilized a customized version of the Stern-Volmer equation. Through this approach, we calculated the static quenching constant, symbolized as ‘V,’ and ascertained it to be 5.5 × 10^9^ mol^-1^ dm^3^. Additionally, we determined the effective quenching radius about the kinetic distance within the sphere of action to be 1.29 × 10^−4^ cm. A reasonable level of agreement with the experimental results was achieved by density functional theory (DFT) in optimizing the excited and ground states of BTMSP dye and by calculating the absorption and emission peaks.

## Supplementary Information

Below is the link to the electronic supplementary material.

Supplementary Material 1

## Data Availability

All data generated or analyzed during this study are included in this article.
