# Supplementary material for: Photophysical behaviour, solvatochromism, and silver nanoparticle-induced superquenching of (E, E)-2,5-Bis(3,4,5-trimethoxystyryl) pyrazine with DFT calculations
Source: Sci Rep. 2025 Sep 29;15:33329. doi: 10.1038/s41598-025-20289-y (PMC12480476; doi:10.1038/s41598-025-20289-y)
Supplement: Supplementary file 1 — Supplementary Material 1 [file 41598_2025_20289_MOESM1_ESM.docx]

**Supporting information for**

**Photophysical Behaviour, Solvatochromism, and Silver Nanoparticle-Induced Superquenching of (E, E)-2,5-Bis(3,4,5-trimethoxystyryl) Pyrazine with DFT Calculations**

Ehab A. Okba^a *^, Yomna M. Hanafi^a^, Tarek A. Fayed^a^, Mahmoud A. S. Sakr^b^, and Samy A. El-Daly^a^

*^a^* *Chemistry Department, Faculty of Science, Tanta University, 31527, Egypt*

*^b^* *Center of Basic Science, Misr University for Science and Technology, 6TH of October City, Egypt.*

Corresponding author: Ehab A. Okba, [ehab_okba@science.tanta.edu.eg](mailto:ehab_okba@science.tanta.edu.eg)


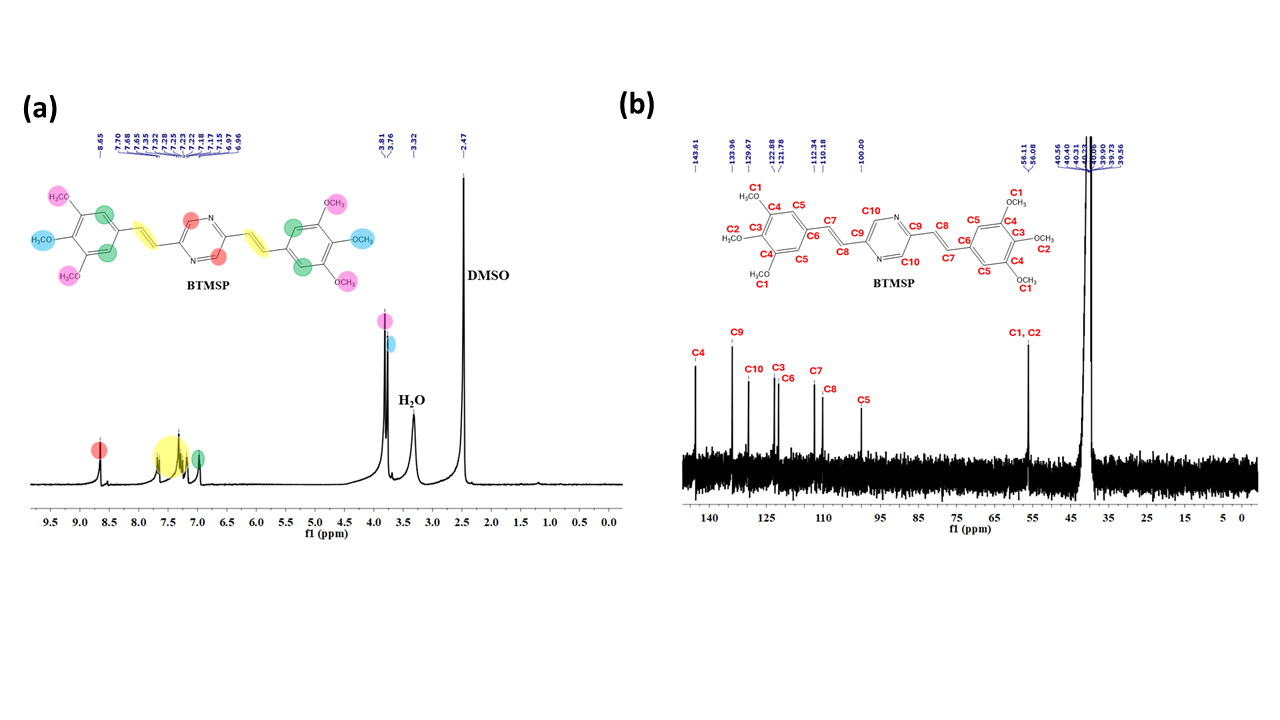


**Fig. S1**(a) ^1^H-NMR spectrum and (b) ^13^C-NMR spectrum of BTMSP


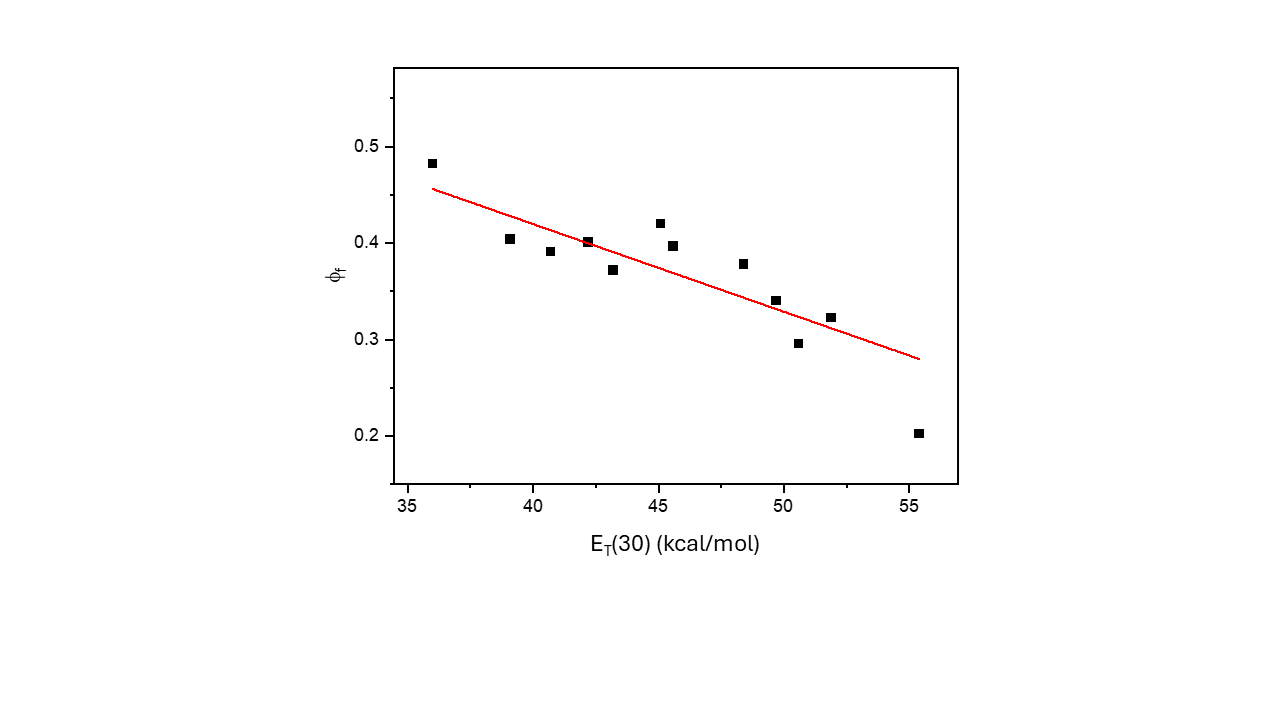


**Fig. S2** - Comparison of BTMSP's fluorescence quantum yield with E_T_ (30) in various solvents

**
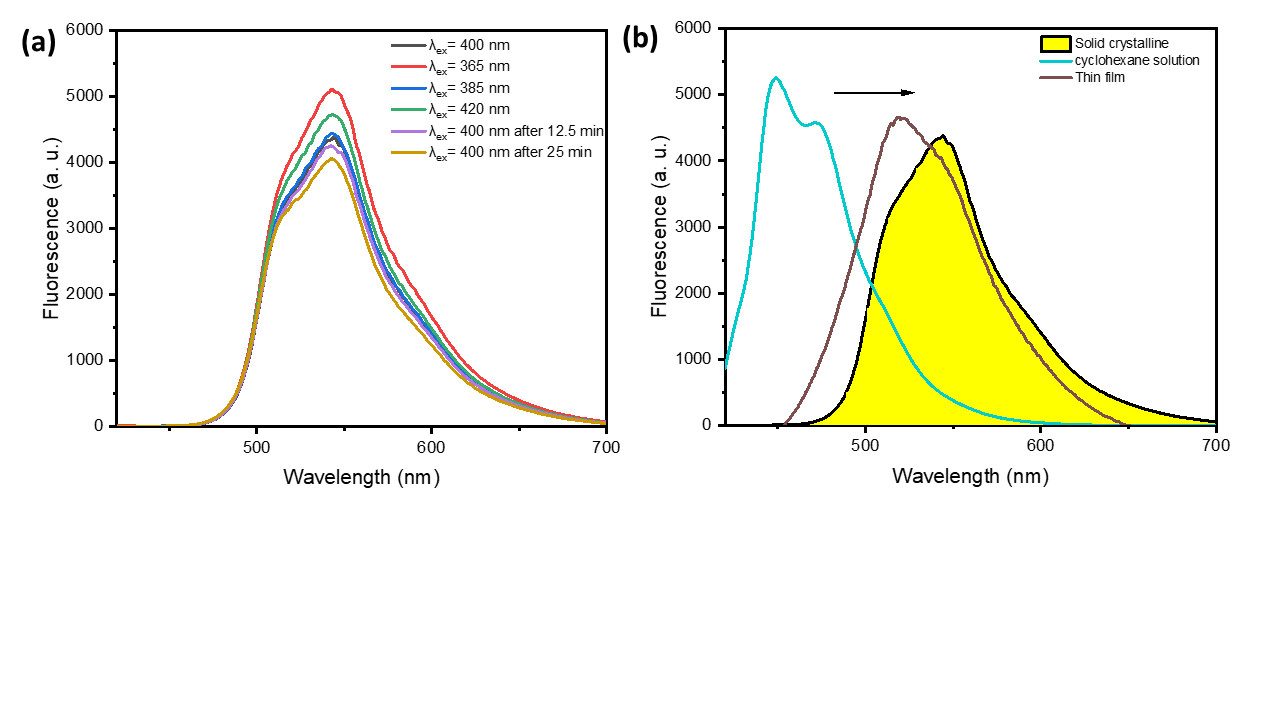
**

**Fig. S3**(a) Fluorescence Spectra of the solid crystal of the BTMSP dye at various excitation wavelengths and (b) Emission spectra of BTMSP in cyclohexane (1 x 10^-5^ mol dm^-3^), thin film, and solid crystalline (λ_ex_ = 400 nm)

The dipole moments of BTMSP dye were determined according to spectral shift using Bakshiev 's and Kawski–Chamma–Vialles equations(S1 and S4), respectively^1-4^. The value of Δμ was found to be 2.53 Debye.

$$\nu_{a}-\nu_{f}=m_{2}. F_{2}\left( \varepsilon,n \right)+constant (S1)$$

where $F_{2}\left( \varepsilon,n \right)=\left[ \frac{\varepsilon-1}{\varepsilon+2}-\frac{n^{2}-1}{n^{2}+2} \right]\left( \frac{2n^{2}+1}{n^{2}+2} \right) (S2)$

$m_{2}=\frac{2\left( \mu_{e}-\mu_{g} \right)^{2}}{hca^{3}} (S3)$

$$\frac{\upsilon_{a}+\nu_{f}}{2}=-m_{3}F_{3}\left( \varepsilon,n \right)+constant (S4)$$

$$F_{3}\left( \varepsilon,n \right)=\frac{1}{2}\left( \frac{2n^{2}+1}{(n^{2}+2)}\left[ \frac{\varepsilon-1}{\varepsilon+2}-\frac{n^{2}-1}{n^{2}+2} \right] \right)+\frac{3}{2}\left[ \frac{(n^{4}-1)}{\left( n^{2}+2 \right)^{2}} \right] (S5)$$

$m_{3}=\frac{2(\mu_{e}^{2}-\mu_{g}^{2})}{hca^{3}} (S6)$

The slopes of the straight lines shown in Fig. S3(a,b) were found to be 2507 cm^-1^ and 4870 cm^-1^, respectively, and their corresponding values, m_2_ and m_3_, were measured. According to equations S7 and S8, the calculations yielded the values of μ_g_ and μ_e_ as 2.02 and 6.32 Debye, respectively. This study demonstrates that the polar nature of the excited state of BTMSP surpasses that of the ground state, primarily due to the redistribution of charge and alteration of geometry that occur in BTMSP during excitation. The Lippert-Mataga equation yields a value of 4.29 Debye for the change in dipole moment (Δμ), which is near the difference between μ_e_ and μ_g_, specifically 4.3 Debye.

$$\mu_{g}=\frac{m_{3}-m_{2}}{2}\left[ \frac{{hca}^{3}}{{2m}_{2}} \right]^{\frac{1}{2}} ( S7 )$$

$\mu_{e}=\frac{m_{3}+m_{2}}{2}\left[ \frac{{hca}^{3}}{{2m}_{2}} \right]^{\frac{1}{2}} (S8)$

According to Eq. (S9), we determined the dye's dipole moment during its transition from the ground to the excited state (μ_12_) in various solvents^5^:

$\mu_{12}^{2}=\frac{f}{4.72 \times{10}^{-7}E_{max}} (S9)$

The absorption maximum energy, denoted as E_max_ in units of cm^-1^, and the oscillator strength, denoted by the symbol (f), are two key parameters in characterising the electronic spectrum. E_max_ signifies the energy at which absorption is maximised, where f measures the number of electrons that are effectively involved in the transition from the ground state to the excited state, which in turn determines the absorption area of the electronic spectrum. Equation (S10) was used to determine the experimental oscillator strength values^6^.

$$f=\frac{4.32}{{10}^{9}}\int\varepsilon\left( \nu\right)d\nu(S10)$$

In this context, "ε" denotes the molar absorption coefficient, which is quantified in dm^3^mol^−1^cm^-1^, and "v" represents the wavenumber, which is quantified in cm^-1^. The μ_12_ values are presented in Table S1.


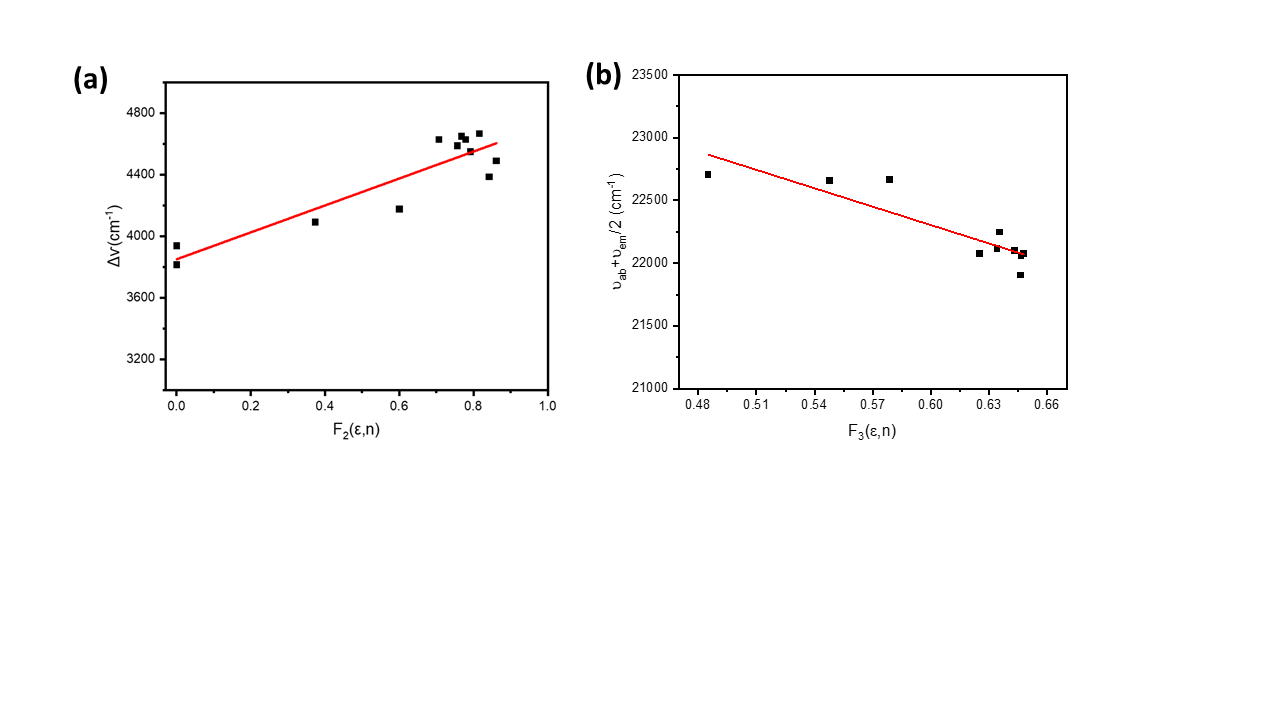


**Fig. S4** - Variation of Stokes’ shift versus F_2_ (ε, n) (a), and F_3_ (ε, n) (b)


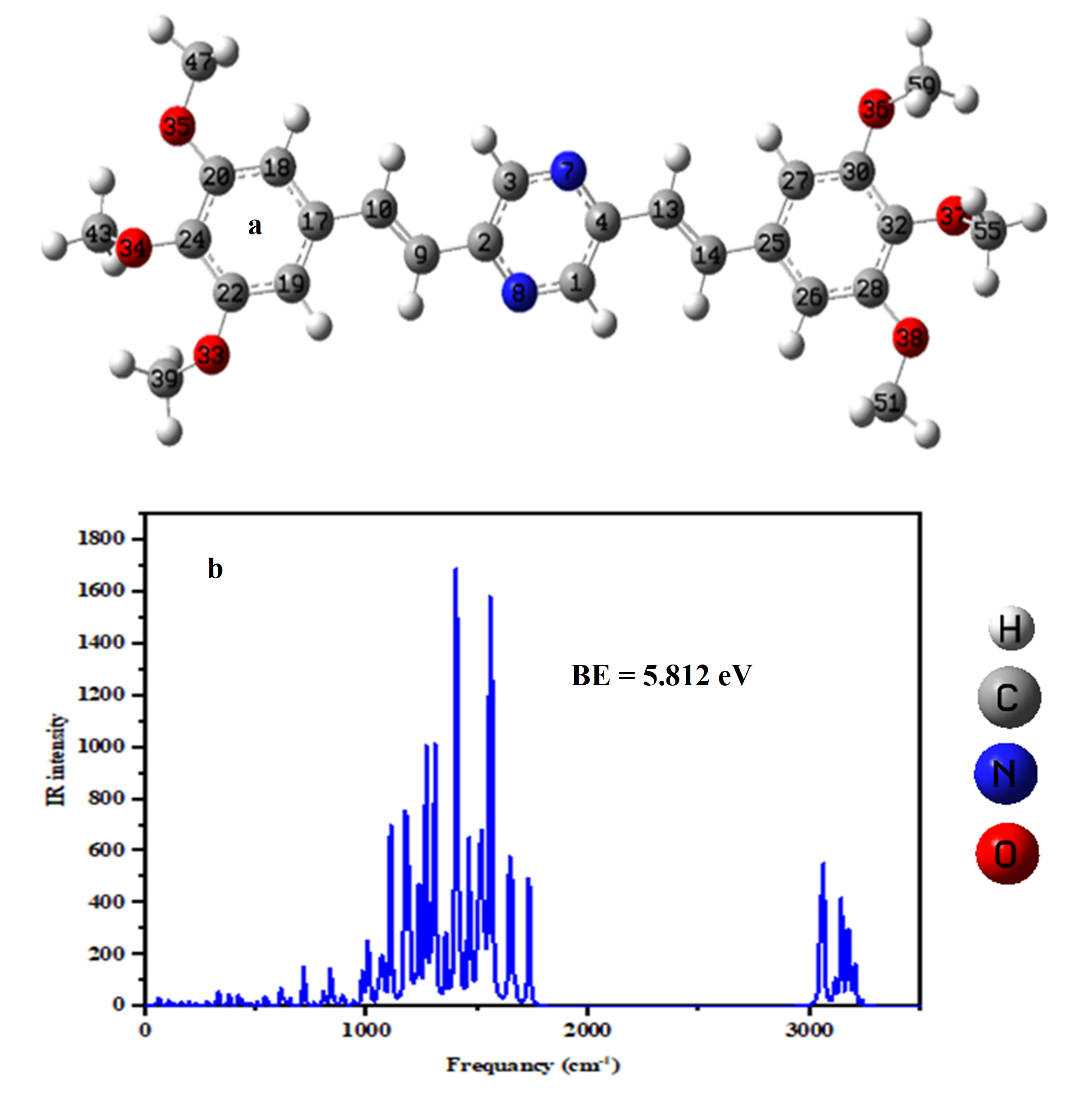


**Fig. S5-** Optimized structure (a) and calculated IR spectrum (b) of BTMSP compound.

**
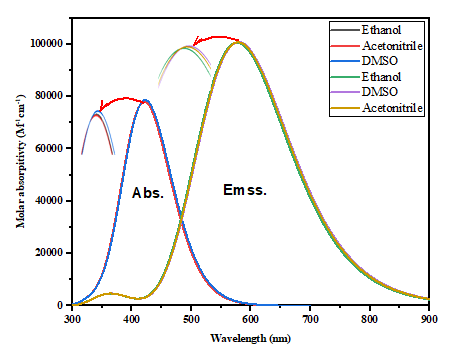
**

**Fig. S6** - Calculated absorbance spectra of BTMSP MS in different solvents.

**Table S1-** **Physical parameters of some solvents;** the oscillator strength f, acidic character α, the solvent polarity function F_1_, F_2_, F_3,_ and transition dipole moment µ_12_

| **Solvents** | **Dielectric constant**  **Ԑ** | **n** | **E_T_(30)**  **kcal/mol** | $\boldsymbol{E}_{\boldsymbol{T}}^{\boldsymbol{N}}$ | **f** | **α** | **F _1_**  (ε, n) | **F _2_**  (ε, n) | **F _3_**  (ε, n) | **µ_12_** |
| --- | --- | --- | --- | --- | --- | --- | --- | --- | --- | --- |
| **1,4 Dioxan** | 2.25 | 1.42 | 36.0 | 0.164 | 0.615 | 0.00 | 0.0253 | 0.0514 | 0.310 | 7.309 |
| **CHCl_3_** | 4.81 | 1.44 | 39.1 | 0.259 | 0.891 | 0.44 | 0.1501 | 0.3739 | 0.485 | 8.733 |
| **CH_2_Cl_2_** | 9.08 | 1.41 | 40.7 | 0.309 | 0.856 | 0.09 | 0.2231 | 0.6007 | 0.578 | 8.559 |
| **n-BuOH** | 17.8 | 1.39 | 49.7 | 0.586 | 0.409 | 0.84 | 0.2673 | 0.7563 | 0.643 | 5.961 |
| **iso-prOH** | 18.3 | 1.37 | 48.4 | 0.546 | 0.502 | 0.76 | 0.2756 | 0.7676 | 0.635 | 6.579 |
| **n-propanol** | 20.1 | 1.38 | 50.6 | 0.617 | 0.655 | 0.84 | 0.2755 | 0.7791 | 0.648 | 7.543 |
| **Acetone** | 20.7 | 1.35 | 42.2 | 0.355 | 0.500 | 0.06 | 0.2875 | 0.7931 | 0.634 | 6.590 |
| **EtOH** | 24.6 | 1.35 | 51.9 | 0.654 | 0.645 | 0.86 | 0.2930 | 0.8166 | 0.646 | 7.485 |
| **MeOH** | 32.6 | 1.32 | 55.4 | 0.762 | 0.797 | 0.98 | 0.3118 | 0.8567 | 0.646 | 8.259 |
| **CH_3_CN** | 36.6 | 1.34 | 45.6 | 0.46 | 0.538 | 0.19 | 0.3064 | 0.8620 | 0.662 | 6.794 |
| **DMF** | 38.2 | 1.42 | 43.2 | 0.386 | 0.735 | 0.00 | 0.2786 | 0.8424 | 0.706 | 7.932 |
| **DMSO** | 47.2 | 1.47 | 45.1 | 0.444 | 0.589 | 0.76 | 0.2661 | 0.8441 | 0.740 | 7.196 |

**Table S2**- Selected optimized structural parameters (bond length in ˚A, bond angle, and dihedral angle in degrees computed for the BTMSP compound in the gaseous phase. For labeling, belong to Fig. S4a.

| Designation | C2-C3 | C2-C9 | C9-C10 | C2-C3-C7 | C20-C18-C17 | C20-C18-C17-C10 | C17-C10-C9-C2 |
| --- | --- | --- | --- | --- | --- | --- | --- |
| Values | 1.402 | 1.464 | 1.339 | 122.810 | 120.462 | 179.731 | 179.823 |

**Table S3 -** The calculated excited state (ES), computational maximum absorbance (λ_ab.Th_) and emission (λ_ems.Th_)wavelength, transition energy (TE), electronic transition (ET), oscillator strength (*f*), transition coefficient (TC) and the experimental maximum absorbance( λ_ab.exp_) and emission (λ_ems.exp_) wavelength for SZ and its derivative materials.

| Solvents | ES | λ_ab.Th_(nm) | TE (eV) | ET | *f* | TC | λ_ab.exp_.(nm) | λ_ems.Th_.(nm) | λ_ems.exp_.(nm) |
| --- | --- | --- | --- | --- | --- | --- | --- | --- | --- |
| Ethanol | 1 | 421.67 | 2.940 | H→L | 1.92 | 0.70522 | 407.00 | 576.49 | 486.00 |
| Acetonitrile | 1 | 422.93 | 2.931 | H→L | 1.933 | 0.70513 | 407.00 | 578.6 | 502.00 |
| DMSO | 1 | 421.23 | 2.943 | H→L | 1.917 | 0.70520 | 418.00 | 579.76 | 497.00 |

**Table S4**- Charge-transfer length (∆r), centroid coordinates of holes and electrons (D), electron-hole overlap (S_r_), hole-electron degree of separation (t), and hole-electron Coulomb attraction energy (E_c_) for BTMSP in S_0_→S_1_/S_2_/S_3_/S_4_/S_5_/S_6_.

| Electronic transitions | D(Å) | Sr(au) | H(Å) | t(Å) | E_c_(eV) | Δr(A) |
| --- | --- | --- | --- | --- | --- | --- |
| S_0_-S_1_ | 0.000 | 0.80306 | 5.229 | -5.032 | 4.021739 | 0.000398 |
| S_0_-S_2_ | 0.000 | 0.67107 | 5.840 | -2.199 | 3.638474 | 0.000083 |
| S_0_-S_3_ | 0.003 | 0.34226 | 3.332 | -2.196 | 4.739669 | 0.000024 |
| S_0_-S_4_ | 0.002 | 0.55208 | 5.822 | -5.046 | 3.234297 | 0.000961 |
| S_0_-S5 | 0.001 | 0.69897 | 5.764 | -1.394 | 3.380277 | 0.000702 |
| S_0_-S_6_ | 0.002 | 0.45306 | 3.823 | -2.939 | 4.293027 | 0.000383 |

References

1 Bilot, v. L. & Kawski, A. Zur theorie des einflusses von Lösungsmitteln auf die elektronenspektren der moleküle. *Zeitschrift für Naturforschung A* **17**, 621-627, doi:<https://doi.org/10.1515/zna-1962-0713> (1962).

2 Kawski, A. On the Estimation of Excited-State Dipole Moments from Solvatochromic Shifts of Absorption and Fluorescence Spectra. *Zeitschrift für Naturforschung A* **57**, 255-262, doi:doi:10.1515/zna-2002-0509 (2002).

3 Ghazy, R., Azim, S. A., Shaheen, M. & El-Mekawey, F. Experimental studies on the determination of the dipole moments of some different laser dyes. *Spectrochimica Acta Part A: Molecular and Biomolecular Spectroscopy* **60**, 187-191, doi:<https://doi.org/10.1016/S1386-1425(03)00205-1> (2004).

4 Liu, L. *et al.* Solvent effect on the absorption and fluorescence of ergone: Determination of ground and excited state dipole moments. *Spectrochimica Acta Part A: Molecular and Biomolecular Spectroscopy* **86**, 120-123, doi:<https://doi.org/10.1016/j.saa.2011.10.016> (2012).

5 Asiri, A. M., El-Daly, S., Alamry, K., Arshad, M. & Pannipara, M. Photoinduced intramolecular charge transfer and photophysical characteristics of (2Z)-3-[4-(dimethylamino) phenyl]-2-(2-methylphenyl) prop-2-ene-nitrile (DPM) in different media. *Journal of Molecular Structure* **1098**, Pp 153-160, doi:<https://doi.org/10.1016/j.molstruc.2015.05.048> (2015).

6 Gordon, P. F. & Gregory, P. *Organic chemistry in colour*. (Springer Science & Business Media, 2012).
